# MMP2 and TLRs modulate immune responses in the tumor microenvironment

**DOI:** 10.1172/jci.insight.144913

**Published:** 2021-06-22

**Authors:** Luciana R. Muniz-Bongers, Christopher B. McClain, Mansi Saxena, Gerold Bongers, Miriam Merad, Nina Bhardwaj

**Affiliations:** 1 Tisch Cancer Institute,; 2 Hematology and Oncology Department, and; 3 Oncological Sciences Department, Icahn School of Medicine at Mount Sinai, New York, New York, USA.

**Keywords:** Immunology, Oncology, Antigen-presenting cells, Melanoma, T cells

## Abstract

The presence of an immunosuppressive tumor microenvironment is a major obstacle in the success of cancer immunotherapies. Because extracellular matrix components can shape the microenvironment, we investigated the role of matrix metalloproteinase 2 (MMP2) in melanoma tumorigenesis. We found that MMP2 signals proinflammatory pathways on antigen presenting cells, and this requires both TLR2 and TLR4. B16 melanoma cells that express MMP2 at baseline have slower kinetics in *Tlr2*^–/–^ *Tlr4*^–/–^ mice, implicating MMP2 in promoting tumor growth. Indeed, *Mmp2* overexpression in B16 cells potentiated rapid tumor growth, which was accompanied by reduced intratumoral cytolytic cells and increased M2 macrophages. In contrast, knockdown of *Mmp2* slowed tumor growth and enhanced T cell proliferation and NK cell recruitment. Finally, we found that these effects of MMP2 are mediated through dysfunctional DC–T cell cross-talk as they are lost in *Batf3*^–/–^ and *Rag2*^–/–^ mice. These findings provide insights into the detrimental role of endogenous alarmins like MMP2 in modulating immune responses in the tumor microenvironment.

## Introduction

The tumor microenvironment (TME) is a complex network of tumor and stromal cells, as well as of signaling molecules that can dampen antitumor immune responses, enabling tumor growth and maintenance. TME composition varies between tumor types but broadly consists of angiogenic vascular cells, cancer-associated fibroblasts (CAFs), immune cells, and an extracellular matrix (ECM) that interact with tumor cells (1, 2). Matrix metalloproteinases (MMPs), a family of extracellular proteinases, are key components in promoting tumor progression through TME modulation. Many members of this family can cleave multiple ECM components and facilitate metastasis. Among these, MMP2 is overexpressed (OE) in many tumors, including melanoma, and high MMP2 levels in tumor or stromal cells are associated with increased tumor invasion and cancer progression, with patients often having poorer survival/prognosis (3). Elevated expression of some MMPs (such as MMP2, MMP1, and MMP13) has been directly correlated with poorer prognosis, and MMP2 — in particular — has been associated with melanoma progression (4–6). Moreover, MMP2 has also been identified as a melanoma-associated antigen that is recognized by tumor-infiltrating lymphocytes (TILs) (7–9), suggesting a dual role in promoting tumor growth as well as in engaging antitumor immunity.

Initially, the role of MMPs in tumorigenesis was presumed to be due to their proteolytic function, but they also have roles that are independent of their catalytic activity (10). In murine macrophages, MMP12 was shown to promote disruption of cellular membranes of phagocytosed bacteria in an enzymatically independent manner (11). Also, MMP14 modulates macrophage function in a protease-independent manner by regulating PI3Kδ signaling (12). We previously identified MMP2 as a cognate ligand for TLR2 signaling in human antigen presenting cells (APCs) that is independent of its catalytic activity. This interaction resulted in DC activation but skewing of T cells toward a Th2 phenotype that is induced through TLR2-mediated OX40L expression and reduced IL-12 production (13). These observations highlight the significance of TLR signaling in modulating immune responses that affect the TME. Indeed, several TLRs have been implicated in mediating pro- or antitumor activity in both tumor and immune cells in the TME (14–18).

Other endogenous TLR ligands, like high motility group box 1 (HMGB1), domain A of fibronectin, fibrinogen, β-defensin 2, soluble hyaluronan, and heparan sulfate also modulate immunity via TLR activation on APCs in homeostasis and in the TME (19–24). Versican (a matrix proteoglycan) is secreted by tumor cells and stimulates metastasis through TLR2 signaling in myeloid cells (25, 26). Similarly, Heat-shock protein 60 signals through TLR2 on tumor cells and promotes lung metastasis in mice (27). Intriguingly, including MMP2, all these molecules mainly target TLR2, -4, or -6, suggesting that activation of TLR2 and -4 via alarmins and subsequent modulation of cells in the TME may be an important process in tumorigenesis and even metastasis. TLR signaling on tumors is a double-edged sword, and while some TLR agonists have shown antitumor effects, many studies reveal a role for TLR activation in promoting tumor growth (14, 17, 27–29). This duality might be tumor specific, where the TME composition influences TLR signaling, but it might also be due to the nature of the TLR ligand, subsequent downstream signaling pathways, and consequent effects on immune cells.

With increasing evidence highlighting the role of MMPs in tumor progression and metastasis, these factors are poised to be an attractive target for therapy. Inhibitors of MMPs have targeted many aspects of their biology, from the inhibition of their synthesis and their interaction with other proteins, to blocking their activity (30); however, synthetic MMP inhibitors (MPIs) failed in clinical trials to improve overall survival in patients with cancer possibly due to their lack of specificity (31–33). There is a need for selective inhibitors that can target individuals sets of MMPs in a specific tumor type, and this could potentially improve clinical outcome in future trials, when used in combination with chemotherapy or immunotherapy approaches. For this, it is imperative to understand the diverse functions of MMPs, which in some cases may diverge from their canonical enzymatic activity.

Based on these observations, we speculated that MMP2 signaling via TLR2 might substantially impact tumorigenesis and metastasis, thereby posing an attractive target for therapy. Interrogating the MMP2-TLR2 axis affords an opportunity to identify new and targeted therapeutic approaches to modulate antitumor immunity in vivo. In this study, we used murine models of cancer to dissect the effects of MMP2 in the regulation of immune responses in the TME.

## Results

### MMP2 signals inflammatory responses through TLR2, TLR4, and MYD88.

**Figure 1:**
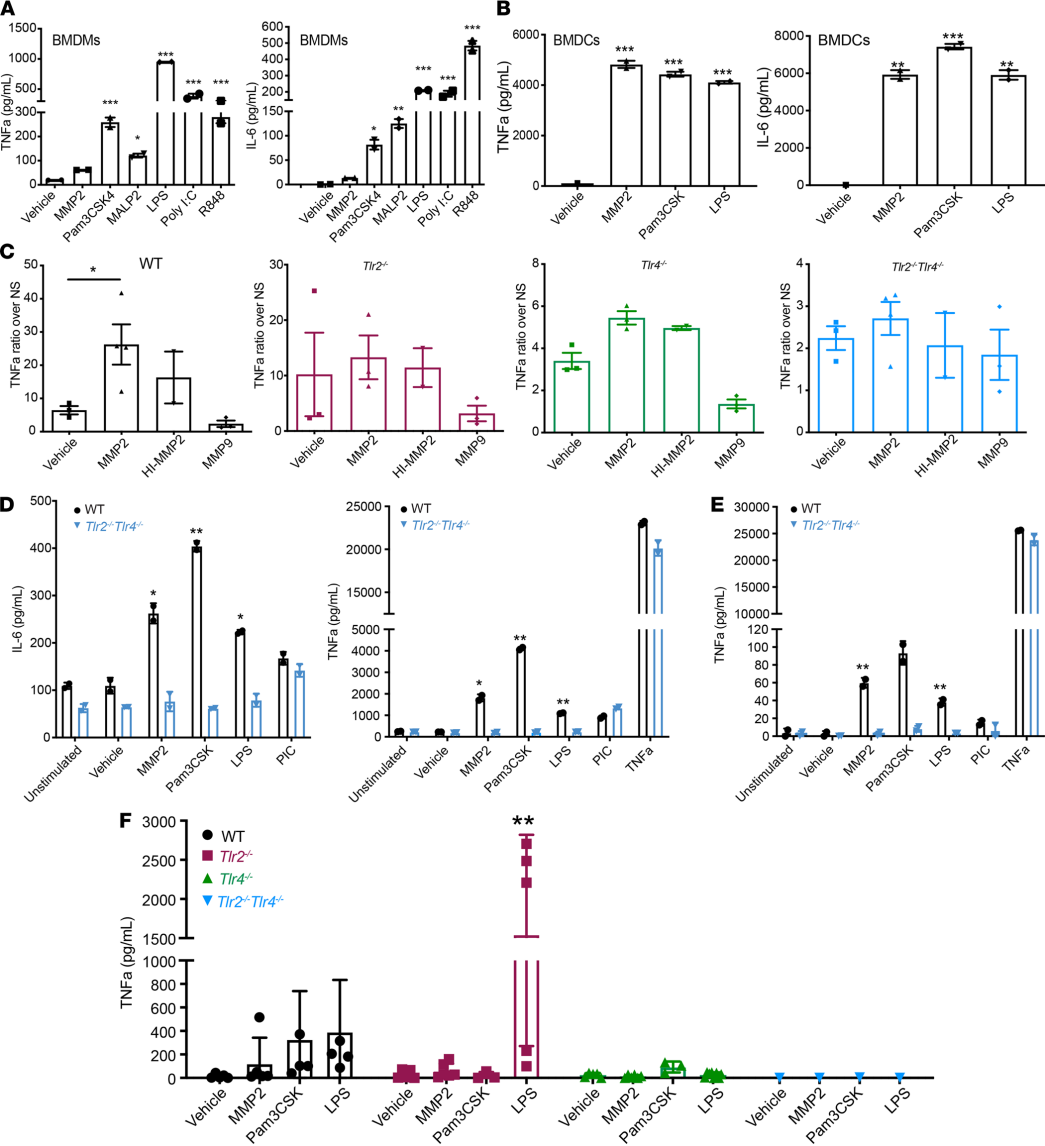
MMP2 signaling is mediated by TLR2 and TLR4. Proinflammatory cytokine secretion by primary APCs and immortalized APC cell lines. Briefly, cells were stimulated with MMP2, MMP9, vehicle control, or TLR agonists overnight. All stimulations were performed with 2 *×* 10^5^ cells per condition in 200 μL volume. Sixteen to 18 hours after stimulation, supernatants were collected and cytometric bead array (CBA) for mouse inflammatory cytokines was performed. (**A** and **B**) BMDMs (**A**) and BMDCs (**B**) responded to stimulation and secreted TNF-α and IL-6. *n* = 2. Data are representative of 2 independent experiments with mean ± SEM. *P < 0.05, **P < 0.01, and ***P < 0.001. One-way ANOVA with Dunnett’s post hoc test. (**C**) TNF-α secretion by Im-MACS from WT and Tlr-deficient mice. Each graph represents the ratio over unstimulated cells. *n* = 2–4. Data are representative of 4 independent experiments with mean ± SEM. *P < 0.05. One-way ANOVA with Dunnett’s post hoc test. (**D** and **E**) TNF-α and IL-6 secretion by primary CD11c^+^ DCs isolated from lung (**D**) and spleen (**E**). *n* = 2. Data representative of 2 experiments. Mean ± SEM. *P < 0.05 and **P < 0.01 versus *Tlr2*^–/–^ *Tlr4^–/–^* cells. Multiple unpaired *t* test with using Holm-Sidak correction for multiple comparisons. (**F**) TNF-α secretion in serum of WT, *Tlr2^–/–^*, *Tlr4^–/–^*, and *Tlr2*^–/–^ *Tlr4^–/–^* mice 3 hours after i.v. injection with Vehicle, MMP2, Pam3CSK4, and LPS. Data are representative of 4 independent experiments with mean ± SEM. **P < 0.01. Two-way ANOVA with Dunnett’s post hoc test.

MMP2 modulates human DCs by TLR2 signaling and IFNAR1 degradation, modifying the DC cytokine response and subsequent T cell priming toward Th2 cells (8). However, the precise mechanism by which MMP2 modulates antitumor immunity is still undefined. To shed light onto this, we investigated MMP2’s function in murine melanoma. To characterize the receptors involved in MMP2 signaling in murine APCs, we stimulated BM-derived APCs with recombinant human MMP2 (rhMMP2) and assessed proinflammatory cytokine secretion. Control stimulants included MMP9 (MMP2’s most related member) and TLR agonists Pam3CSK4 (TLR1/2), MALP2 (TLR2/6), LPS (TLR4), Poly I:C (TLR3), and R848 (TLR7). We previously ensured that our sources of MMPs were free of endotoxin contamination (8, 13). Primary BM-derived macrophages (BMDM) and BMDC secreted TNF-α and IL-6 (Figure 1, A and B) in response to MMP2 but not to vehicle control or MMP9. As expected, the BMDMs and BMDCs secreted proinflammatory cytokines in response to the canonical TLR ligands (Pam3CSK4, MALP2, LPS, Poly I:C, and R848), with only BMDCs lacking response to Poly I:C (Figure 1B).

Next, immortalized mouse BMDM (Im-Macs) lacking TLRs or downstream signaling adaptors (WT, *Tlr2*^–/–^ *Tlr9*^–/–^, *Tlr4*^–/–^, *Tlr2*^–/–^ *Tlr4*^–/–^, *Myd88*^–/–^, *Trif*^–/–^ *Myd88*^–/–^, *Trif*^–/–^, and *Tram*^–/–^) were stimulated by TLR agonists and controls (Figure 1C and Supplemental Figure 1; supplemental material available online with this article; https://doi.org/10.1172/jci.insight.144913DS1). MMP2 required both TLR2 and -4 for signaling, and cells that lacked either of these TLRs were significantly reduced in their ability to respond to MMP2 and secrete TNF-α. Furthermore, TNF-α secretion was almost completely abrogated in *Tlr2*^–/–^ *Tlr4*^–/–^ Im-Macs (Figure 1C). In line with our previous published data (8), MMP2’s enzymatic activity was dispensable as heat-inactivated (HI) MMP2-induced cytokine secretion in WT but not Tlr-deficient Im-Macs (Figure 1C). Notably, none of the cells responded to TLR3 stimulation by Poly I:C, indicating the lack of TLR3 expression upon BMDM immortalization (Supplemental Figure 1A). MYD88, but not TRIF, is involved in MMP2 signaling, since dual *Myd88^–/–^* and *Trif^–/–^ Myd88^–/–^* cells — but not *Trif^–/–^* cells — lost their response to MMP2 as compared with WT cells (Supplemental Figure 1B).

Since TLR2 and -4 were both necessary for MMP2 signaling, we evaluated the responsiveness of APC derived from double KO (DKO) *Tlr2*^–/–^ *Tlr4*^–/–^ mice. CD11c^+^ DCs isolated from the lungs and spleen of WT and DKO mice were stimulated with MMP2 or controls ex vivo. DCs from WT but not DKO mice responded to MMP2 by secreting proinflammatory cytokines (Figure 1, D and E).

Finally, the physiologic relevance of MMP2 signaling via TLR2 and -4 in vivo was confirmed by injecting MMP2 and controls i.p. in WT or TLR-deficient mice and assessing the proinflammatory cytokine response in the serum 3 hours later. TNF-α secretion was evident in the serum of WT but not in *Tlr2*^–/–^, *Tlr4*^–/–^, or DKO mice (Figure 1F). Altogether, these data indicate that the signaling complex involved in MMP2 signaling in mAPCs is composed of at least TLR2, TLR4, and MyD88 and is required for a response to MMP2 both in vitro and in vivo.

### MMP2 binds to TLR2 and TLR4.

**Figure 2:**
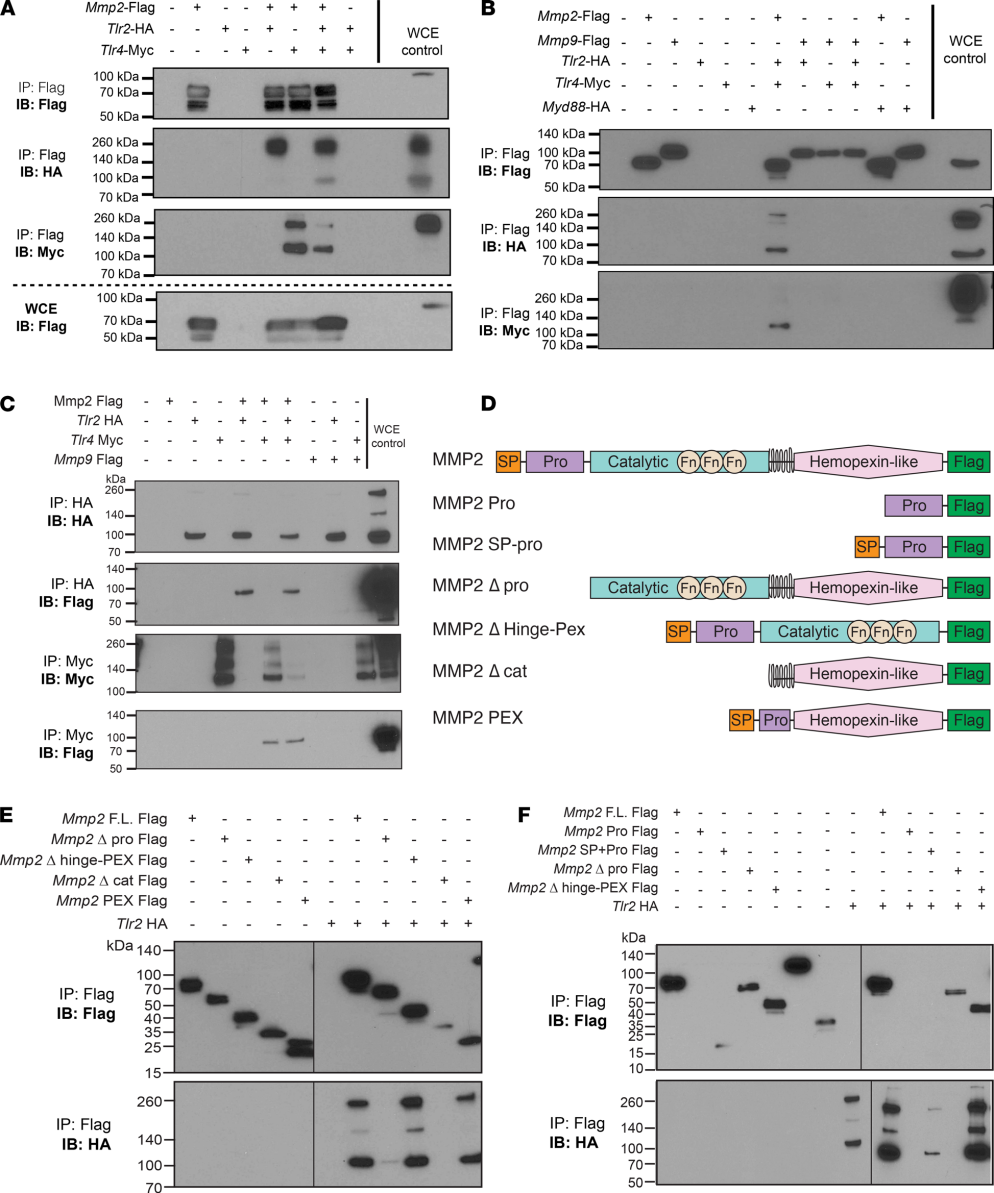
MMP2 binds and precipitates with TLR2 and TLR4. (**A** and **B**) HEK293T cotransfection followed by Flag pull-down using magnetic beads. *Mmp2*-Flag was cotransfected with *Tlr2*-HA, *Tlr4*-Myc, or both plasmids (**A**). *Mmp9*-Flag cotransfection with Tlr-plasmids or *Myd88*-HA plasmid was also evaluated (**B**). (**C**) Reverse co-IP of *Mmp2*-Flag or *Mmp9*-Flag cotransfected with *Tlr2*-HA, *Tlr4*-Myc, or both plasmids. HA or Myc pull-down using anti-HA agarose or Anti–c-MYC agarose beads. (**D**) Schematic of the different MMP2 constructs used in the co-IP to identify the binding domain. (**E** and **F**) Different MMP2 domains were deleted or expressed alone and tested for co-IP with *Tlr2*-HA. Black line indicates where membrane ends/was cut. All transfections were performed using Lipofectamine 3000, and protein was extracted 20–24 hours after transfection. IP, immunoprecipitation; IB, immunoblotting; WCE, whole-cell extract; Δ, deletion.

To confirm that TLR2 and -4 interact with MMP2 and to explore if other signaling mediators participate in the complex, we performed co-IP experiments. HEK293T cells were cotransfected with murine *Mmp2* Flag and murine *Tlr2* HA — or murine *Tlr4* Myc — and, as shown previously (13), MMP2 bound and precipitated with TLR2. We also determined that TLR4 coprecipitated with MMP2 (Figure 2A). When all 3 plasmids were cotransfected, both TLR2 and -4 precipitated with MMP2, suggesting no competition for binding (Figure 2A). TLR2 and -4 binding was specific to MMP2, as they did not precipitate with MMP9 (Figure 2B) and MMP2 did not coprecipitate with *MyD88* HA (Figure 2B). To confirm specificity, we performed reverse IP by pulling down HA (TLR2) or Myc (TLR4) and probing for MMP2 (Figure 2C). Both TLR2 and -4, but not MYD88, bound MMP2. This suggests that TLR2 and -4 may form a heterodimer that binds to MMP2 and initiates a signaling complex that recruits MYD88 for signaling. Altogether, our data confirm that MMP2 associates with TLR2 and -4, thereby accounting for the inflammatory responses seen in Figure 1. See complete unedited blots in the supplemental material.

### MMP2 binds with TLR2 and TLR4 via its SP-Pro domains.

We next sought to identify which MMP2 domain binds the TLRs. Plasmids with different *Mmp2* domains expressing a Flag-tag (Figure 2D) were generated and co-IP with full-length *Tlr2*-HA was performed. The signal peptide (SP) and Pro domains of the MMP2 protein were required for binding with TLR2 (Figure 2E). Using a plasmid expressing only SP + Pro domains, we observed that they, alone, were sufficient for precipitation with TLR2 (Figure 2F). Due to technical difficulties, the constructs with only SP or only Pro domains could not be evaluated (Figure 2F).

Interestingly, the hemopexin domain, which is involved in canonical roles of the MMP2, was dispensable for binding. Only when the hemopexin plasmid also included SP and Pro domains (*Mmp2*-PEX construct) was it able to precipitate TLR2 (Figure 2F). In summary, our data suggest that MMP2, through the presence of SP and Pro domains, binds TLR2 and -4 and forms a complex.

### MMP2 expression in murine melanoma.

We next evaluated MMP2 expression in melanoma using B16 F1 and F10 murine melanoma cells. We assessed MMP2 and TLR expression in 3 F1 cell lines (F1, F1 YFP-expressing, and F1 OVA-expressing) and one B16 F10 line. MMP2 was detected in concentrated supernatants (Supplemental Figure 2A) and in whole-cell extract (WCE; Supplemental Figure 2B) of all B16 cell lines tested. However, B16 cells lacked TLR2 expression by Western blot (WB; Supplemental Figure 2B). Upon stimulation with MMP2, MMP9, and TLR agonists (Pam3CSK4, MALP2, LPS, Poly I:C, and R848), B16 F1 cell lines did not respond to MMP2 or any TLR agonists, whereas control BMDMs responded as expected (Supplemental Figure 2, C and D). Thus, while F1 tumor cells express MMP2, they lack the ability to respond to TLR activation or MMP2 stimulation. These data exclude the possibility that autocrine or paracrine production of MMP2 will impact TLR2/4-dependent signaling pathways in tumor cells. See complete unedited blots in the supplemental material.

To determine if MMP2 is produced in B16 tumors in vivo, we sorted CD45^+^, YFP^+^ tumor, and YFP^–^ stromal cells from tumors and evaluated gene expression by reverse transcription PCR (RT-PCR). *Mmp2* mRNA was expressed by both tumor and stromal cells, but not infiltrating CD45^+^ cells (Supplemental Figure 2E). YFP^+^ tumor cells expressed neither *Tlr2* nor *Cd14* and expressed little *Tlr4*, indicating that, in vivo, they are also unlikely to signal upon TLR2-TLR4 ligation (Supplemental Figure 2, E and F). Altogether, these data indicate that, while our melanoma cells can produce MMP2, due to lack of signaling machinery, they cannot directly respond to it in an autocrine or paracrine manner. However, MMP2 released by tumor cells may directly affect APCs or other cells within the TME that express *TLR2* and *TLR4*.

### Smaller tumor growth and kinetics in Tlr2^–/–^ Tlr4^–/–^ mice.

**Figure 3:**
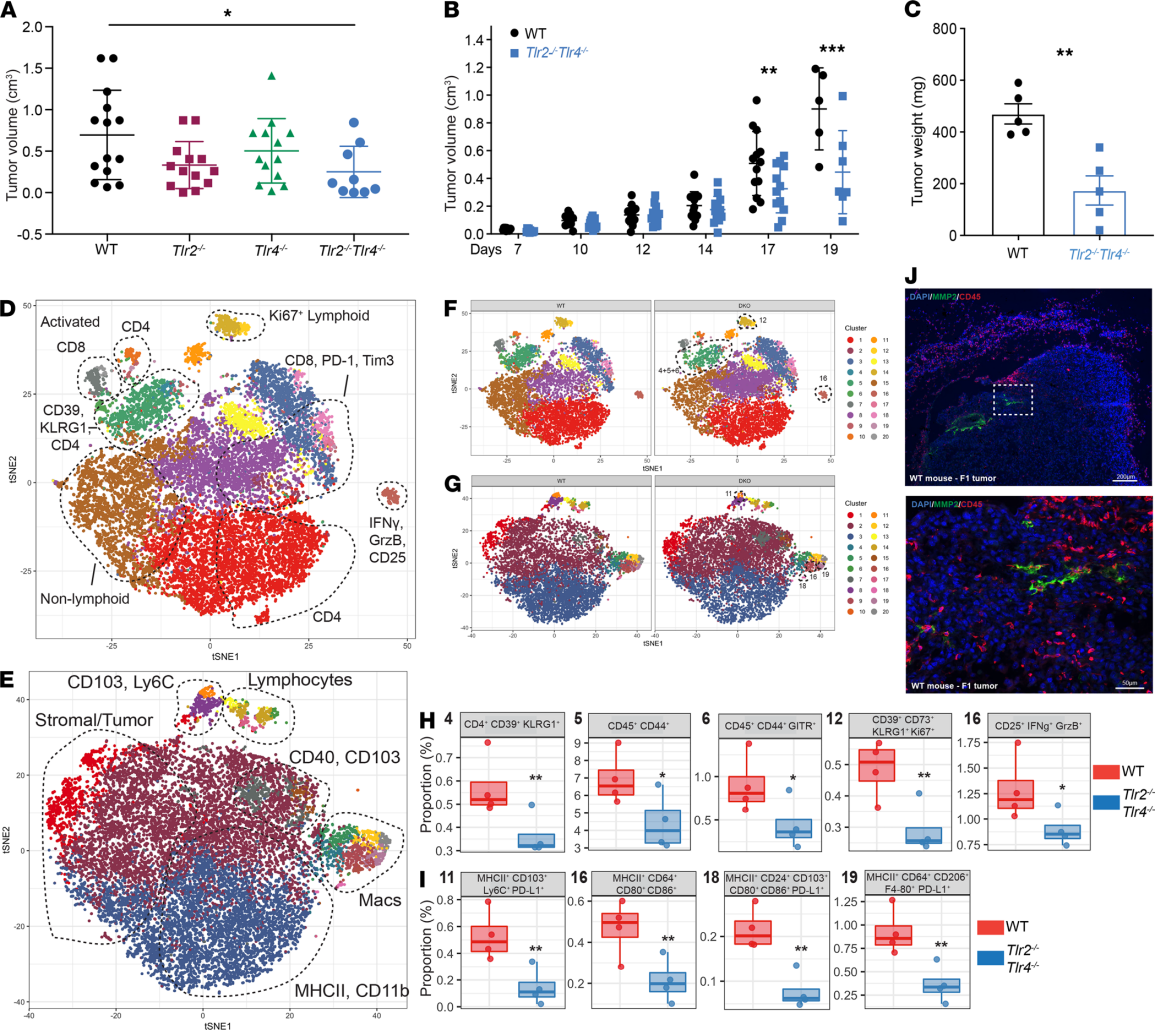
Smaller tumor growth and differential tumor microenvironment in *Tlr2^–/–^ Tlr4^–/–^* mice. In total, 3 *×* 10^5^ B16 F1 cells were injected s.c. into WT or *Tlr-*deficient mice, and tumors were analyzed 15–19 days later. (**A**) Tumor volume in WT, *Tlr2*^–/–^, *Tlr4^–/–^*, and *Tlr2*^–/–^ *Tlr4^–/–^* mice at day 15. Data are representative of 4 independent experiments with mean ± SEM. *P < 0.05. Two-way ANOVA with Dunnett’s post hoc test. (**B**) Tumor growth kinetics between WT and *Tlr2*^–/–^ *Tlr4^–/–^* mice during the course of 19 days. (**C**) Tumor weight (mg) at day 18–19. (**A**–**C**) Data are representative of 5 independent experiments with mean ± SEM. **P < 0.01 and ***P < 0.001. Two-way ANOVA with Sidak’s correction for multiple comparisons. (**D**–**I**) CyTOF analysis of lymphoid and myeloid panels in the TME at day 19 between WT and *Tlr2*^–/–^ *Tlr4^–/–^* mice. viSNE plot of immune, stromal, and tumor cell clusters present in the lymphoid (**D**) and myeloid (**E**). Comparison between WT and *Tlr2*^–/–^ *Tlr4^–/–^* mice in the lymphoid (**F**) and myeloid (**G**), highlighting differentially expressed clusters (dotted lines). Statistically significant clusters from the lymphoid (**H**) and myeloid (**I**) are identified. Data are representative of 2–4 mice with mean ± SEM. *P < 0.05 and **P < 0.01. *P* values were adjusted using FDR. Statistical analysis was performed using binomial generalized linear mixed-effects model (GLMM). (**J**) IF staining of MMP2 (green), CD45 (red) and DAPI (blue). Scale bars: 200 μm for the top panel, 50 μm for the bottom panel.

To determine whether *Tlr2* and *Tlr4* signaling is required for tumor progression, we monitored tumor incidence and growth up to 19 days following B16 F1 injection in WT, *Tlr2*^–/–^, *Tlr4*^–/–^, and DKO mice. There was a significant delay in tumor growth with reduced tumor incidence and weight in DKO mice versus WT, while single *Tlr2*^–/–^ and *Tlr4*^–/–^ mice presented an intermediate phenotype (Figure 3, A–C). Although the total number of cells isolated from DKO tumors was smaller, we observed no major differences in the percentage of immune cell infiltrates within total CD45^+^ cells by FACS (Supplemental Figure 3A). Thus, the differences in tumor growth observed are likely due to differences in the quality or function of specific immune cell subsets rather than their quantity.

To gain a more comprehensive idea of the differences between WT and DKO, tumors were analyzed by CyTOF, using panels distinguishing lymphocytes or myeloid and stromal cells. Twenty population clusters were identified on each panel (Figure 3, D and E). The clusters could be separated between myeloid, lymphoid, stromal, and tumor cell compartments based on distinct marker analysis and clustering (Supplemental Figure 3, B and C). Comparison between mouse WT and DKO mice was also performed to separate clusters differentially (Figure 3, F and G). Statistically significant clusters were identified (Figure 3, H and I, and Supplemental Figure 3, D and E). In the lymphocyte panel, we noted 5 clusters (clusters 4, 5, 6, 12, and 16), which were statistically significantly decreased in tumors of DKO origin when compared with WT ones (Figure 3F). The clusters 4, 5, 6, and 12 include populations of T cells expressing CD4, CD39, CD73, KLRG1, and Ki67 — all markers found to be expressed on Tregs (34, 35). Cluster 16 includes a population of CD25^+^ cells that express IFN-γ, granzyme B, and low levels of FoxP3, Ki67, and CD44. Based on these markers, this cluster might be composed of cytolytic T cells or a subset of NKT cells (36) (www.immgen.org). Altogether, we observed differences within the subpopulations in the T cell compartment in tumors of DKO mice that support the differences observed in the tumor growth (Figure 3H and Supplemental Figure 3).

With respect to myeloid cells, there was a significant decrease of 4 clusters in tumors from DKO mice as compared with WT — namely, clusters 11, 16, 18, and 19 (Figure 3, G and I). Based on marker expression (CD103, CD80, CD86, CD64, F4-80, and others), these cells were mainly composed of DCs, macrophages, and granulocytes all sharing PD-L1 expression (Figure 3I and Supplemental Figure 3C). PDL1 expression by APCs in the TME and in the tumor-draining lymph nodes (LNs) can inhibit T cell activation and can be detrimental for tumor growth (37), and the decrease of PDL1^+^ myeloid cells observed in tumors from DKO mice might explain the slower tumor kinetics. Finally, MMP2 expression was assessed by IF staining of tumors from WT mice. MMP2-expressing cells were found proximal to CD45^+^ cells (Figure 3J), suggesting an interaction between hematopoietic cells that express TLR2 and -4 and MMP2^+^ tumor or stromal cells (13).

Altogether, these results indicate that the TME in DKO mice is characterized by a reduction in Treg-like cells and PDL1^+^ APCs, suggesting a less immunosuppressive and immunoexhaustive environment underlying the smaller tumor size and delayed kinetics.

### Expression of TLR2 and TLR4 in the hematopoietic compartment aids tumor development.

We created BM chimeras to dissect the role of TLR2 and -4 in stromal versus hematopoietic compartments for tumor growth. WT or DKO mice were sublethally irradiated and injected i.v. with WT or DKO BM cells (Supplemental Figure 4A). There were no major differences in the ability of the BM cells to reconstitute blood (Supplemental Figure 4, B and C) or tissues such as lungs or spleen of WT mice (Supplemental Figure 4D).

Following BM reconstitution, mice received B16 cells, and tumor progression was monitored. Tumor growth and incidence was substantially reduced in WT mice with DKO BM as compared with WT mice that received WT BM (Supplemental Figure 4, E and F). Donor BM, from WT and DKO origin, reconstituted recipients with the same efficiency, and CD45^+^ cells were found in tumors at similar proportions. However, because of smaller tumor sizes, the absolute number of reconstituted CD45.2^+^ cells was lower in recipients receiving DKO BM (Supplemental Figure 4G and data not shown). In complementary experiments, smaller tumors were observed in DKO recipients that received DKO BM when compared with WT BM, indicating a role for hematopoietic TLRs in modulating B16 tumor growth in vivo (Supplemental Figure 4H). A role for nonhematopoietic cell TLR2 and -4 expression in supporting tumor growth was also noted, as tumors in DKO mice that received WT BM were smaller than tumors in WT mice that received WT BM (Supplemental Figure 4H).

Analysis of the TME by FACS did not reveal major differences in the proportion of recruited immune cell populations in any of the recipient groups (Supplemental Figure 4, I and J). No differences in IFN-γ– and TNF-α–single positive CD4^+^ T cells were noted. However, there was a slight increase (although not significant) in the proportion of IFN-γ^+^TNF-α^+^CD4^+^ T cells in tumors of WT mice that received DKO BM, when compared with WT BM (Supplemental Figure 4K), suggesting a skewing toward Th1 phenotype. These results indicate that the quality and function of the immune cell infiltrates in mice receiving DKO BM are altered and may account for the observed tumor growth differences. To confirm this, a more detailed dissection of the T cell quality, activation state, and exhaustion phenotype of the T cells from these tumors will be needed.

In summary, the expression of *Tlr2* and *Tlr4* in the TME is important for the promotion of tumor growth, and when both of these receptors are absent, growth is compromised. Furthermore, the expression of *Tlr2* and *Tlr4* in both hematopoietic and stromal compartments appears to support *Mmp2*-driven tumor growth.

### Overexpression of Mmp2 in B16 cells accelerates tumor growth and promotes a protumorigenic TME.

**Figure 4:**
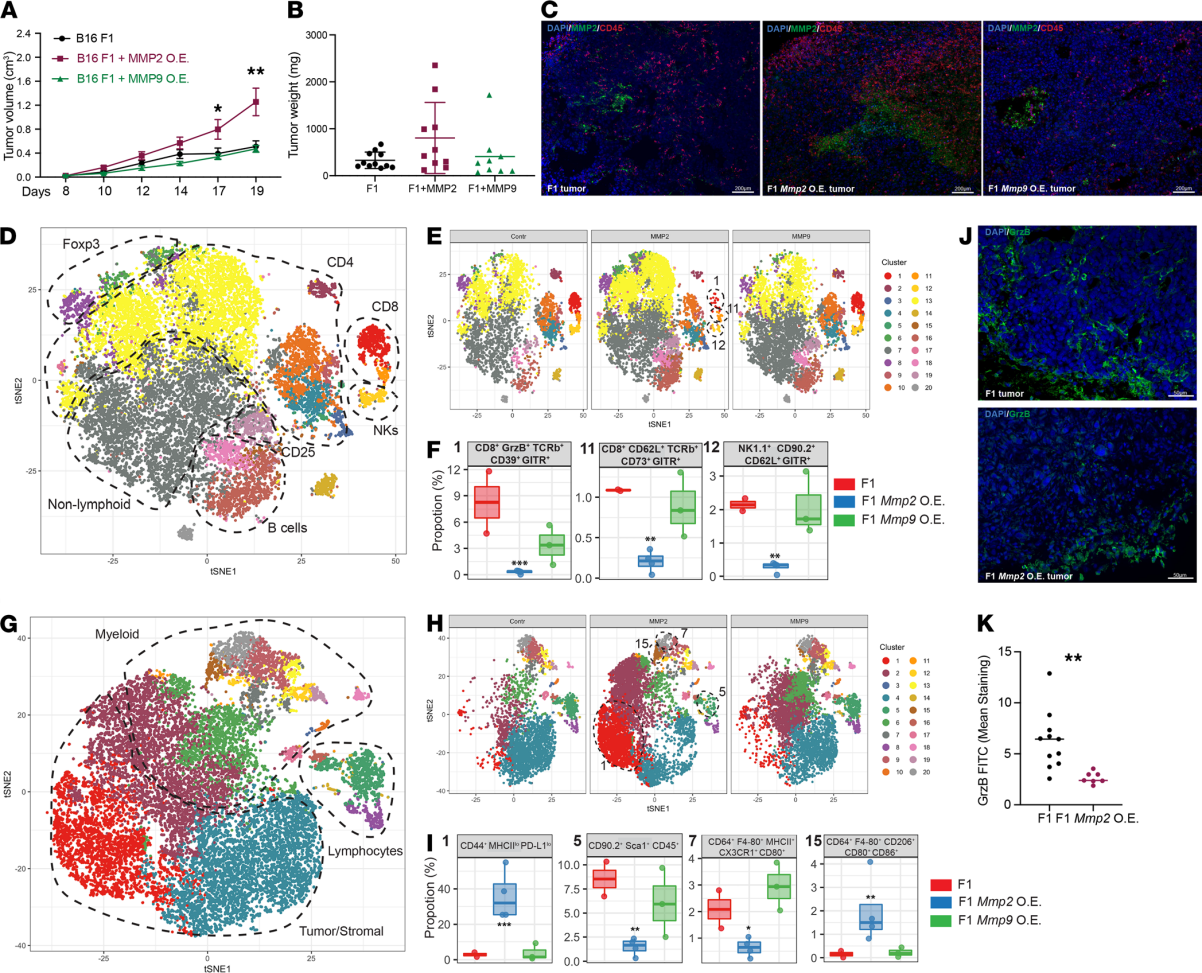
*Mmp2* overexpression in B16 cells promotes melanoma tumor growth. *Mmp2* and *Mmp9* were OE in B16 F1 cells and injected into mice. (**A** and **B**) Tumor kinetics was measured up to 20 days. Tumor volume (**A**) and weight (**B**) are displayed. *n* = 8–10 mice per group. Data are representative of 5 independent experiments with mean ± SEM. *P < 0.05 and **P < 0.01. Two-way ANOVA with Dunnett’s post hoc test. (**C**) Immunofluorescence (IF) staining for MMP2 (green), CD45 (red), and DAPI (blue). Scale bars: 200 μm. (**D**–**F**) CyTOF analysis of lymphoid panel. viSNE plot of immune, stromal, and tumor cell clusters present in the tumors at day 19 (**D**) identified with aid of single marker expression in all samples. Population comparison between F1 and F1 *Mmp*-OE tumors (**E**) were identified, and clusters differentially expressed between the different groups of tumors were identified (**F**). (**G**–**I**) CyTOF analysis of Myeloid panel. viSNE plot of immune, stromal, and tumor cell clusters present in the tumors at day 19 (**G**) identified with aid of single marker expression in all samples. Population comparison between F1 and F1 MMP-OE tumors (**H**) were identified, and clusters differentially expressed between the different groups of tumors were identified (**I**). Data are representative of 2–4 mice with mean ± SEM. *P < 0.05, **P < 0.01, and ***P < 0.001. *P* values were adjusted using FDR. Statistical analysis was performed using binomial generalized linear mixed-effects model (GLMM). (**J**) IF of granzyme-B (green) and DAPI (blue) in F1 tumors (top) and F1 *Mmp2*-OE tumors (bottom). Scale bars: 50 μm. (**K**) Quantification of GrzB staining. *n* = 8–12 sections with mean ± SEM. **P < 0.01. Student’s *t* test.

The precise contribution of *Mmp2* in tumor cells was investigated through modulation of their MMP2 expression. First, *Mmp2* and *Mmp9* were stably OE in B16 F1 cells (Supplemental Figure 5A). The OE cells secreted active MMPs (Supplemental Figure 5B) and had similar growth rates (Supplemental Figure 5D) in vitro. In vivo, the *Mmp2*-OE cells grew faster and bigger than the control tumors (Figure 4A). At days 18–20, tumor weight was measured (Figure 4B) and tumors were processed. IF confirmed the overexpression and distribution of MMP2 in the B16-OE tumors (Figure 4C) and displayed a pronounced recruitment of CD45^+^ cells into the TME. See complete unedited blots in the supplemental material.

CyTOF was performed with lymphoid and myeloid/stromal cell panels. FlowSOM analysis revealed 20 population clusters (Supplemental Figure 6A). Cells from *Mmp2* and *Mmp9*-OE tumors clustered separately from each other (Supplemental Figure 6B). Applying a graph-based clustering (t-distributed stochastic neighbor embedding [tSNE]), we identified clusters of lymphoid, myeloid, and stromal/tumor origin (Figure 4, D and G) based on the expression of single markers (Supplemental Figure 6, C and E). B cells, NK cells, Foxp3^+^ T cells, CD4^+^ T cell subsets, and CD8^+^ T cells were identified (Figure 4D and Supplemental Figure 6C). The tSNE plots for the 3 different tumor groups. F1, F1 *Mmp2-*OE, and F1 *Mmp9*-OE were generated (Figure 4E) to highlight the differential expression of the individual clusters (Supplemental Figure 6D). A statistically significant decrease in 3 clusters (1, 11, and 12) was apparent when *Mmp2*-OE tumor infiltrates were compared with WT infiltrates (Figure 4F). Clusters 1 and 11 are composed of CD8^+^ T cells positive for cytotoxicity marker granzyme B and markers for activation and tissue resident memory (Trm) or “stemness,” as recently described (38, 39). Based on the ectonucleotidase expression (e.g., CD39), these could also be effector T cells differentiating toward exhaustion (40–44). Additionally, these CD8^+^ T cells also expressed GITR, which while mostly known for its expression in Tregs, can also be expressed in activated CD4^+^ and CD8^+^ T cells, provide costimulation for CD8^+^ T cell activation, and promote CD8^+^ T cell clonal expansion (45–47). Finally, cluster 12 included NK cells that express GITR and CD62L, indicating a possible mature polyfunctional NK cell population (48, 49) (Figure 4F and Supplemental Figure 6D). Altogether, *Mmp2*-OE tumors appear to be selectively depleted of stem-like CD8^+^ T cells and NK cells, both important for tumor control when activated (50–53).

With respect to myeloid cells, the tSNE plots for F1, F1 Mmp2-OE, and F1 Mmp9-OE tumors were generated (Figure 4H) to highlight the differential expression of the individual clusters (Supplemental Figure 6E). Four clusters (1, 5, 7, and 15) were statistically different in *Mmp2*-OE tumors versus controls (Figure 4I and Supplemental Figure 6F). Cluster 1 was increased in *Mmp2*-OE tumors and composed of cells high in CD44 and low in MHCII, PD-L1, and Sca-1 (Ly6a) expression. Cluster 15, composed of M2-like macrophages expressing CD11c, CD64, F4-80, and CD206, was also selectively increased in the *Mmp2*-OE tumors. Two clusters were significantly smaller in *Mmp2*-OE tumors: clusters 5 and 7. Cluster 7 is composed of M1-like macrophages that express CD64, F4-80, MHCII, and CX3CR1. Cluster 5 was characterized by CD90.2^+^Sca1^+^CD45^+^, with lower levels of CD44, CD69, and CD86 expression, indicating they could be CD8^+^ memory T cells or NKT cells (54, 55). Because the myeloid panel lacks CD4 or CD8, it is still unclear whether cluster 5 was composed of CD4 or CD8 subsets.

These results were confirmed by IF where *Mmp2*-OE tumors showed decreased granzyme B^+^ cell infiltration (Figure 4, J and K). Taken together, the results reveal that the TME of *Mmp2* overexpressing tumors in WT mice is enriched in M2-like macrophages but reduced in tumor-reactive cytotoxic CD8^+^ T cells and polyfunctional mature NK infiltration, which together would compose a protumorigenic TME.

### Depletion of Mmp2 in B16 cells reduces tumor growth and promotes T cell proliferation in the TME.

**Figure 5:**
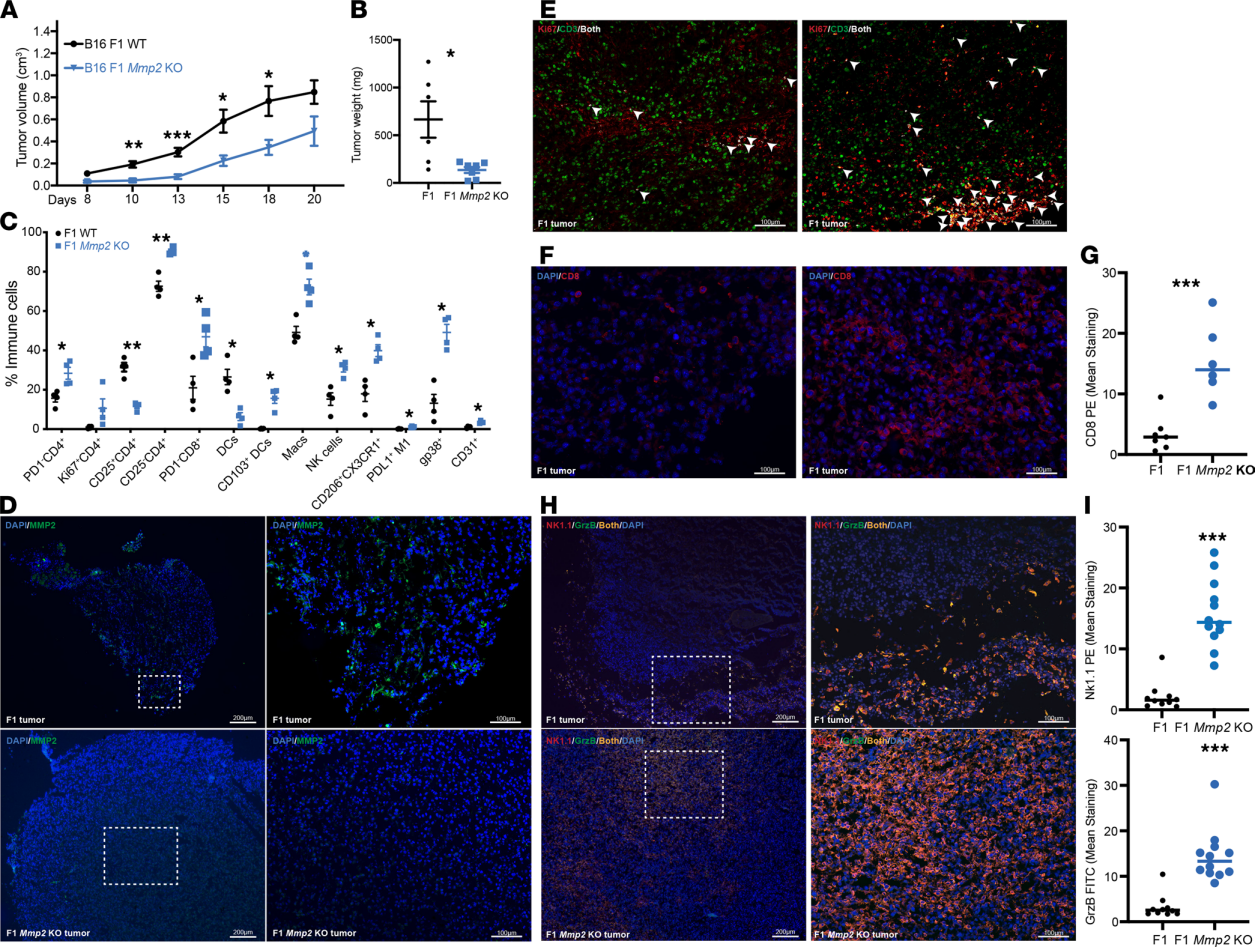
*Mmp2* depletion in B16 cells impairs melanoma tumor growth. The levels of *Mmp2* in the B16 was modulated by CRISPR KO systems. In total, 3 *×* 10^5^ B16 F1 or F1 *Mmp2*-KO cells and tumors were measured up to 20 days. (**A** and **B**) Tumor volume (**A**) and weight (**B**) are displayed. Data are representative of 5 independent experiments with mean ± SEM. *n* = 8–10 mice per group. *P < 0.05, **P < 0.01, and ***P < 0.001. Multiple *t* tests with Holm-Sidak correction for multiple comparisons. (**C**) FACS analysis on day 19 shows changes in hematopoietic cell infiltration. Data are representative of 3 independent experiments with mean ± SEM. *n* = 4 mice per group. *P < 0.05 and **P < 0.01. Multiple *t* tests with Holm-Sidak correction for multiple comparisons. (**D**–**I**) Immunofluorescence (IF) stainings. (**D**) IF staining for MMP2 (green) and DAPI (blue). F1 control tumors on the top panel and F1 *Mmp2*-KO controls on the bottom panel. Scale bars: 200 μm on left panels and 100 μm on right panels. (**E**) IF colocalization (white) of CD3^+^ T cells (green) with Ki67 (red) in *Mmp2*-KO tumors. Colocalization is shown is white arrowheads. Scale bars: 100 μm. (**F**) IF staining of CD8 (red) and DAPI (blue). Scale bars: 100 μm. (**G**) Quantification of the CD8 staining. Data representative of 2 experiments. *n* = 6. mean ± SEM. ***P < 0.001. Student’s *t* test. (**H**) IF staining of NK1.1 (red), granzyme B (green), and DAPI (blue) infiltrates in the tumor bed. F1 tumors on top panels and F1 *Mmp2*-KO tumors on the bottom panels. Scale bars: 200 μm on left panels and 100 μm on right panels. Colocalization is shown in orange. (**I**) Quantification of NK1.1 and granzyme-B staining. Data are representative of 2 experiments. *n* = 6. mean± SEM. ***P < 0.001. Student’s *t* test.

We next determined whether depleting tumors of *Mmp2* would alter growth kinetics. *Mmp2*-KO B16 cells were generated using the CRISPR-Cas9 RNP system and confirmed by a T7 endonuclease activity assay (Supplemental Figure 5C). WT or *Mmp2*-KO tumors grew at similar rates in vitro (Supplemental Figure 5D). In vivo, however, *Mmp2*-KO tumors were significantly smaller (Figure 5A) and had reduced weights (Figure 5B) when compared with WT tumors. Significant increases in immune populations with antitumor properties, such as CD4^+^CD25^–^ non-Tregs, CD103^+^ cross-presenting DC and M1-like macrophages (CD206^–^) were evident by FACS in the *Mmp2*-KO tumors (Figure 5C). Additionally, a trend in increased NK cells and PD-1^–^CD8^+^ T cells was observed. IF analysis confirmed the lack of MMP2 expression in *Mmp2*-KO tumors growing in vivo (Figure 5D, lower panels). An increase in T cell proliferation shown by colocalization of CD3 and Ki67 (Figure 5E, white arrowheads) and higher levels of CD8^+^ T cell infiltration were also confirmed in the *Mmp2*-KO versus WT tumors (Figure 5, F and G). Finally, greater numbers of NK cells with associated granzyme B infiltrated the tumor bed in *Mmp2*-KO tumors. In contrast, NKs in WT controls localized mainly at the edges of the tumors (Figure 5, H and I). The gating strategy for the flow cytometry is described in Supplemental Figure 7. Altogether, the lack of *Mmp2* in F1 tumors promoted a higher tumor control, and the immune landscape was characterized by T cell proliferation (indicated by Ki67 expression) and infiltration of cytotoxic T cells, NK cells, cross-presenting CD103^+^ DCs, and M1 macrophages.

The experiments with *Mmp2*-OE and *Mmp2-*KO tumors satisfactorily complement each other, indicating that *Mmp2* promotes immune dysregulation in the TME, by failing to recruit cytotoxic T cells or the cross-presenting CD103^+^ DC and enhancing the infiltration of detrimental M2-like macrophages.

### Tlr2 and Tlr4 are required for the accelerated growth of Mmp2-OE tumors.

To assess the role of host *Tlr2* and *Tlr4* in *Mmp2-*OE and *Mmp2-*KO tumor kinetics, we compared tumor growth in the *Tlr2*^–/–^ *Tlr4^–/–^* DKO mice. The accelerated growth of *Mmp2-*OE tumors observed in WT recipients was lost in DKO recipients (Supplemental Figure 8A), and tumor weights were similar (Supplemental Figure 8B). There was, however, a higher tumor incidence in *Mmp2-*OE tumors (Supplemental Figure 8C). These results suggest that the rapid growth of *Mmp2-*OE tumors relies on TLR2 and -4 expression in host cells. On the other hand, *Mmp2* KO tumors had impaired growth (Supplemental Figure 8A) with smaller weights in DKO mice (Supplemental Figure 8B). Overall, the incidence of *Mmp2-*KO tumors was similar to WT controls but lower than *Mmp2-*OE tumors in DKO hosts (Supplemental Figure 8C).

FACS analysis of the TME of the DKO recipients revealed no major differences in the immune cell landscape between tumors from WT, OE, or KO cells (Supplemental Figure 8, D–I). *Mmp2* overexpression in tumor cells is therefore likely to impact other pathways beyond *Tlr2/4* signaling that contribute to tumor growth — e.g., degradation of ECM proteins to modify the TME architecture or degradation of IFNAR1 in immune cells (APCs or other cell types) as we previously showed (8). Together, these results— along with those in Figures 1 and 3, and Supplemental Figure 4 — emphasize an important role for host derived *Tlr2* and *Tlr4* for potentiating tumor growth.

### Accelerated tumor growth in Mmp2-OE cells partially depends on cDC1s and lymphoid cells.

**Figure 6:**
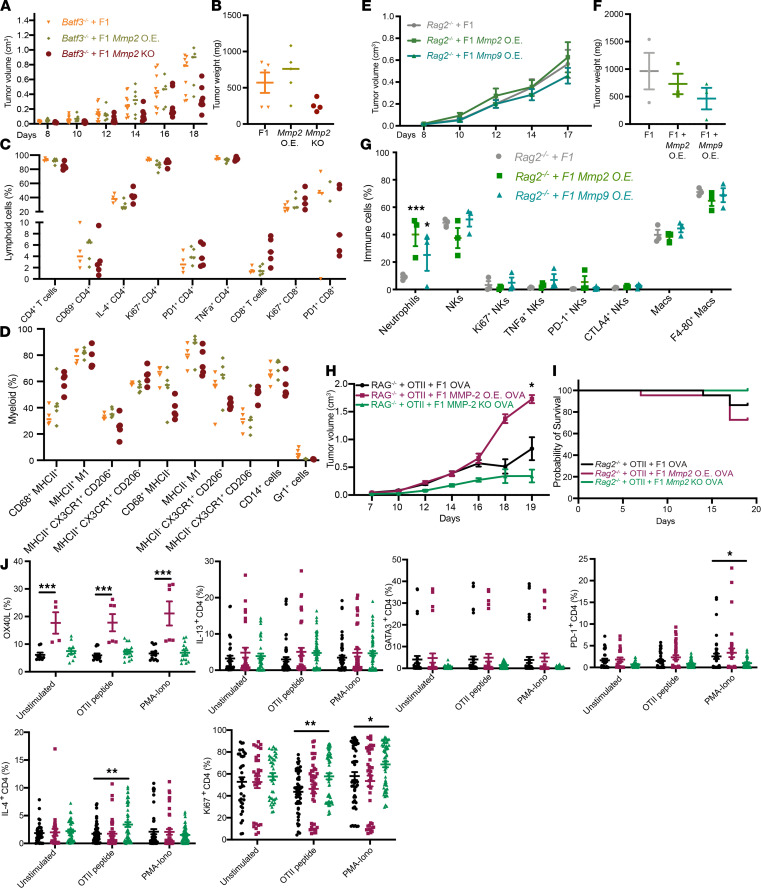
MMP2 signaling in B16 TME involves BATF3 DCs and lymphoid cells. The roles of lymphoid cells and BATF3 DCs were evaluated by using *Rag2^–/–^*and *Batf3^–/–^* mice, respectively. Mice were s.c. injected with 3 *×* 10^5^ B16 F1, F1 *Mmp2*-OE, *Mmp9*-OE, or F1 *Mmp2*-KO cells, and tumors were measured up to 20 days. (**A**–**D**) Tumor growth comparison in *Batf3^–/–^* mice. Tumor volume (**A**) and weight (**B**) are displayed. Data are representative of 3 experiments with mean ± SEM and 8–10 mice per group. One-way ANOVA with Dunnett’s post hoc test. FACS analysis on day 18 shows changes in hematopoietic cell infiltration for lymphoid (**C**) and myeloid cells (**D**). Data are representative of 2 experiments with mean ± SEM and 4 mice per group. Two-way ANOVA with Dunnett’s post hoc test. (**E** and **F**) Tumor growth comparison in *Rag2^–/–^* mice. Tumor volume (**E**) and weight (**F**) are displayed. (**G**) FACS analysis on day 18 shows changes in hematopoietic cell infiltration. Data are representative of 2 experiments with mean ± SEM and 5–7 mice per group. (**H** and **I**) Tumor growth comparison in *Rag2^–/–^* mice transferred with OTII cells and B16 OVA-OE tumors. Tumor volume (**H**) and survival curve (**I**) are displayed. (**J**) FACS analysis of ex vivo stimulated CD4^+^ T cells from tumors at day 18. Data are representative of 2 independent experiments with mean ± SEM. *n* = 4–6 mice per group. Student’s *t* test, *P < 0.05, **P < 0.01, and ***P < 0.001.

Our studies identified a mechanism by which *Mmp2* modulates antitumor activity: ligation of TLR2/4 on APCs promotes tumor infiltration of myelosuppressive populations while reducing tumor infiltrating CD4^+^ and CD8^+^ T cells, NK cells, and CD103^+^ DCs. To firmly establish a role for DCs, we evaluated the growth of F1, *Mmp2-*OE, and *Mmp2-*KO tumors in *Batf3*^–/–^ mice, which lack the cross-presenting cDC1 (56), including the CD103^+^ DCs (Figure 6A). In the absence of BATF3^+^ cells, the growth advantage of *Mmp2-*OE tumors was lost. In contrast, *Mmp2-*KO tumors showed significantly delayed growth (Figure 6, A and B). These results support a role for BATF3^+^ DCs in *Mmp2*-driven tumor growth but also suggest that non-BATF3^+^ APCs expressing TLR2 and TLR4 also support tumor growth. Indeed, in mice, both cDC1s (BATF3, CD103^+^ subset) and cDC2s (CD11b^+^ subset) express several TLRs, including TLR2 and -4 (57). FACS analysis did not reveal significant differences in the recruitment of immune cells into the TME (Figure 6, C and D).

To evaluate the contribution of lymphoid cells toward *Mmp2*-driven tumor growth, F1, F1 *Mmp2-*OE, and F1 *Mmp9*-OE cells were injected into *Rag2*^–/–^ mice, which lack T and B cells (58), and tumor growth was monitored. Tumor size and growth patterns were comparable between different cell lines (Figure 6, E and F). Analysis of the TME revealed no major differences in any immune cell infiltrates analyzed (Figure 6G). Altogether, they indicate that *Mmp2* signals through both BATF3^+^ DCs and T cells to enhance tumor growth, and in the absence of *Mmp2,* this dependency is lost.

We previously observed that MMP2-stimulated APCs prime T cells toward a Th2 phenotype (13), which is typically detrimental toward tumor control (7, 59, 60). To determine whether MMP2 modulates its protumorigenic effects through T cell skewing, we activated OTII CD4^+^ T cells (OVA-specific) and transferred them into *Rag2*^–/–^ mice (i.v.). Two days later, we injected F1 cells that OE OVA in the presence or absence of *Mmp2* (F1 OVA, F1 *Mmp2-*OE OVA, or F1 *Mmp2* KO OVA cells) and followed tumor growth kinetics. We theorized that MMP2 would skew T cells toward a Th2 phenotype and potentiate tumor growth. Indeed, larger tumors were observed in *Rag2*^–/–^ mice that received F1 *Mmp2-*OE OVA cells versus F1 OVA cells. Moreover, F1 *Mmp2-*KO OVA tumors grew to a smaller degree than the other 2 cell lines (Figure 6H). Mice receiving F1 *Mmp2-*OE OVA cells had higher mortality rates than mice receiving F1 OVA cells (Figure 6I). CD45^+^ lymphocytes were isolated from tumors and stimulated in vitro with OTII peptide. Overall, CD45^+^ cells from F1 *Mmp2-*OE OVA tumors had higher levels of OX40L expression and increased fractions of IL-13^+^CD4^+^ T cells; this correlates with a Th2 phenotype (Figure 6J). Both F1 OVA and F1 *Mmp2-*OE OVA tumors had more CD25^+^ and PD-1^+^ CD4^+^ T cells than the F1 *Mmp2-*KO OVA tumors. And in all 3 tumors, CD4^+^ T cells were able to proliferate at similar rates (shown by Ki67; Figure 6J). However, all the changes observed in Figure 6J are minimal; thus, a more in-depth phenotypic characterization (i.e., CyTOF or single-cell RNA sequencing [single-cell RNA-seq]) of the Th subsets is necessary to confirm a preference toward a Th2 phenotype in the context if *Mmp2* OE.

In summary, OE of *Mmp2* in melanoma cells promotes tumor growth and depends upon the presence of cDC1s and lymphocytes as the absence of either of these cell populations abrogates the growth advantages. Moreover, *Mmp2* OE skews priming of tumor antigen-specific T cells toward a Th2 phenotype. These data align with our previous results in human cells, in which MMP2 modulated APC priming of T cells toward a potentially protumorigenic phenotype (13).

## Discussion

We previously identified several unique qualities of *MMP2* in human tumors. First, *MMP2* is a bona fide melanoma-associated self-antigen that is recognized by both CD4^+^ and CD8^+^ T cells present in patient TILs. Second, MMP2 is an alarmin that signals via TLR2 to activate human DCs. Third, MMP2 primes T cells toward a deleterious Th2 phenotype through the inhibition of IL-12 and induction of OX40L (8, 9, 13). In this context, OE of *MMP2* in melanoma would be predicted to have deleterious outcomes on the immune system, in addition to promoting tumor growth and invasion through modulation of the stromal architecture.

To address how *MMP2* mechanistically modulates tumor growth, we adopted murine models of melanoma. We confirmed that the APC response to MMP2 stimulation requires *Tlr2* expression — as well as, surprisingly, *Tlr4* expression — in a *Myd88*-dependent manner. The involvement of both *TLR2* and -4 in the response to MMP2 was confirmed in *Tlr2*^–/–^ *Tlr4^–/–^* mice. While the lack of both receptors completely abrogated the response to MMP2, a more modest phenotype was observed in either *Tlr2^–/–^* or *Tlr4^–/–^* mice, which could indicate a compensatory role of each TLR in the absence of the other.

Co-IP experiments confirmed that MMP2 binds both TLR2 and -4 independently. MMP2 was shown to specifically and directly bind TLR2 in surface plasmon resonance experiments with high affinity (K_D_ = 3.22 *×* 10^–8^ M) (13). Our co-IP experiments, however, indicate that the MMP2-TLR4 interaction may not depend upon TLR2, as TLR4 precipitates with MMP2 even in the absence of TLR2. CD14 is mostly known to mediate LPS transfer to a TLR4–MD-2 complex, but it was also shown to bind Pam_3_CSK_4_ (a TLR1/2 ligand) and direct it to TLR2 (61). Therefore, MMP2 binding to the TLR2-TLR4 complex might also rely on CD14 or other adaptors that are still unknown. Our data are also consistent with other reports that TLR2 and -4 form heterocomplexes and that MYD88 is critical for this heterodimer formation (62–64).

To dissect which domain of MMP2 binds to TLR2, we cloned different Flag-tagged *Mmp2* plasmids (Figure 2) and identified MMP2 SP and Pro domains as necessary for the binding. These results are surprising, as secreted MMP2 lacks the SP and Pro domains (cleaved off before secretion). One possibility is that, in the context of tumors (like melanoma), the full-length MMP2 protein might be secreted via exosomes from tumor or stromal cells. It is also possible that tumor cells release full length MMP2 as they undergo necrosis. Indeed, MMP2 (and other MMPs) has been found in exosomes derived from immune, tumor and stromal cells in the TME (65–67).

Since TLR2 and -4 can undergo internalization into endosomes upon activation, full-length MMP2 may also bind TLRs in this intracellular compartment. TLR4 internalizes into endosomes upon LPS stimulation (68, 69), and TLR2 can be internalized into endosomes via MyD88, TRAM, and IRF7 or in a clathrin/dynamin-dependent endocytosis process (70, 72). MMP2 has been detected in endocytic membranes in association with calveolin proteins (73, 74). It can also associate with thrombospondin 2 and be taken up by low-density lipoprotein–related receptor into endosomes similar to the endocytosis of MMP2-TIMP2 complexes (75, 77). The noncanonical role of MMP2’s SP-Pro domains in TLR2 signaling, thus, differs from MMP2’s main role in degradation of ECM protein via its catalytic domain or in migration via its hemopexin domain (78). This could represent an additional way of regulating MMP2 activity in the context of tumor establishment and progression.

Since our results highlighted the requirement of TLRs in MMP2 signaling, we compared B16 growth in WT versus *Tlr2*^–/–^ *Tlr4^–/–^* DKO mice. We observed a significant delay in tumor growth and size in DKO mice, accompanied by changes in TME composition favoring a less regulatory environment. Our results corroborate other studies of tumor growth in *Tlr2-* and *Tlr4*-deficient mice, suggesting that *Tlr* deficiency mitigates tumor progression (25). Indeed, neutralization of TLRs in B16 melanoma with lung metastasis, Lewis Lung Carcinoma, and head and neck squamous cell carcinoma was shown to inhibit tumor growth and metastasis and tumor outcome (27, 29, 79).

Our B16 F1 cells lack TLR2 and -4 expression, ensuring that any response to MMP2 in the TME observed is due to host immune or stromal cells. The lack of TLR2 and -4 expression is not unusual, as B16 cells from different sources can vary in their expression of TLRs and adaptor molecules, with some groups reporting expression of TLR2, while others report the opposite (14, 27). BM chimera experiments, to distinguish the role of the hematopoietic versus the nonhematopoietic compartment, indicated that TLR2 and -4 expression within the former was required for optimal tumor growth. Others have also shown that tumor-derived TLR2 ligands (like versican) induced DC dysfunction in the B16 TME, and in this context, lack of TLR2 or versican improved DC activation and subsequently T cell responses against the (26).

We confirmed a protumorigenic role for MMP2 as its overexpression exacerbated tumor growth. *Mmp2*-OE tumors had fewer infiltrating cytotoxic cells (CD8^+^ T cells and NKs) and more M2-like macrophages. Additionally, there seemed to be a reduction in Trm cells. Trm cells have an important role in tumor control (both human and mouse tumors), and their presence is correlated with better clinical outcome (50–53). Thus, the reduction of this population in *Mmp2*-OE tumors highlights the detrimental role that overexpression of *Mmp2* has in the melanoma TME and explains the overt tumor growth.

In contrast, *Mmp2-*KO tumors had slower growth kinetics and smaller size when compared with WT tumors. This was accompanied by an increase in CD4^+^ and CD8^+^ T cells, M1 like macrophages, NK cells, and CD103^+^ DC, with an increase in proliferating CD4^+^ T cells (Ki67^+^). These data are also consistent with patterns of delayed tumor growth in *Tlr2^–/–^* mice observed by others, suggesting that the disruption of detrimental TLR2-4 signaling in the tumor likely contributes to the control of tumor growth and restoration of immune cell function (25, 26). In a prostate cancer model using *Mmp2^–/–^* mice, reduced liver metastasis and angiogenesis and increased survival was noted (80). We also observed an increase in gp38^+^ stromal cells in *Mmp2*-KO tumors. Gp38 is expressed by CAFs, stromal cells, and CD31^+^ endothelial cells. Gp38^+^ stroma can serve as a barrier to prevent tumor cell invasion into the surrounding tissue and is correlated with improve prognosis (81). An increase in CD31^+^ endothelial-like stromal cells was described in other *Mmp2*-deficient tumors, suggesting that their increased presence in *Mmp2*-deficient tumors contributes toward tumor control (82).

We investigated the role of relevant immune cell populations in controlling MMP2-driven tumor growth. BATF3 cDC1s (CD8α^+^ in lymphoid tissues and CD103^+^ in nonlymphoid ones) were key in mediating MMP2 activity in tumors, as, in their absence, *Mmp2*-OE tumor growth was reduced. DC dysfunction due to TLR2 signaling in B16 melanoma has been previously described (26). Interestingly, *Mmp2*-KO tumors also show a delay in growth kinetics in *Batf3*^–/–^ mice when compared with B16 F1 controls. One possibility for this phenotype is that other DC subsets or APCs substitute for cDC1 activity in the absence of MMP2 and promote tumor control. Indeed, cDC2 are able to cross-present tumor antigens and induce CD4^+^ T cell responses (including a Th2 phenotype) not relying on BATF3 DCs (83–86). It should be pointed out that *Batf3*^–/–^ mice are not a perfect model for depletion of these cross-presenting DCs, as *Batf3* deficiency is not critical for CD8α^+^ DC generation (87, 88) or for cross-presentation to certain antigens to occur (89). Altogether, the effects in *Batf3*^–/–^ mice highlight that cDC1s are negatively affected by OE of *Mmp2* and, in their absence, tumor growth in response to *Mmp2* OE is compromised.

The role for lymphocytes in mediating MMP2 modulatory effects was highlighted using *Rag*-deficient mice. The absence of lymphocytes reduced the overt tumor growth of *Mmp2*-OE cells and reconstitution with CD4^+^ T cells (OTII^+^) partially rescued the phenotype. Consistent with our previous data (13), *Mmp2* OE led to the skewing of tumor-specific T cells toward a Th2 phenotype. Other groups have also shown similar associations between MMP2 and tumor progression and invasion (3, 90, 91).

In tumors OE MMP2, we propose that tumor resident DCs, and possibly other APCs such as TAMs, (92), are negatively modulated by MMP2 via their expression of TLR2 and -4. This in turn leads to skewed T cell priming, reduced CTL and NK cell activation, and inefficient tumor control. Another possibility is that stromal cells, which can express TLR2 and -4 (92), are similarly modulated by MMP2, indirectly affecting T cell responses, by acting on APCs or other immune cells. In this regard, stromal cells can produce inflammatory as well as immune suppressive factors. Signaling of TLR4 on mesenchymal stem cells in the TME, for instance, leads to suppression of NK cell cytotoxicity and MCP1 secretion, both associated with promotion of breast cancer cell migration (93).

Altogether, our results reveal complexities underlying MMP2 signaling in mice, in which TLR2, TLR4, and MYD88 are necessary. MMP2 expression in melanoma promotes tumor growth in a TLR2- and TLR4-dependent manner that requires APCs and T cells. These findings help pave the way for a potential new generation of MMP2 inhibitors, which could target the biding of MMP2 protein to TLRs and disrupt this deleterious MMP2 signaling in the context of tumors.

## Methods

Supplemental Methods are available online with this article.

### Mice

WT C57BL/6J (JAX:000664), CD45.1 (B6.SJL-*Ptprc^a^ Pepc^b^*/BoyJ, JAX:02014), *TLR2^–/–^* (B6.129-Tlr2^tm1Kir^/J, JAX:004650), *TLR4^–/–^* (B6.B10ScN-Tlr4^lps–del^/JthJ, JAX:007227), *Rag2^–/–^* (B6(Cg)-Rag2^tm.1Cgn^/J, JAX:008449), and OTII (B6.Cg-Tg[TcraTcrb]425Cbn/J, JAX:004194) mice were purchased from The Jackson Laboratory. *Tlr2^–/–^ Tlr4^–/–^* mice were generated by crossing *TLR2^–/–^* and *TLR4^–/–^* mice. *Batf3^–/–^* (C.129S-Batf3^tm1Kmm^/J, JAX:013756) mice were obtained via Miriam Merad (Precision Immunology Institute and Tisch Cancer Institute, Icahn School of Medicine at Mount Sinai, New York, New York, USA).

### Cell lines and cell culture

#### Murine Im-Macs.

Immortalized macrophages were used as previously described (94). Briefly, macrophages were immortalized by infecting BM progenitors with oncogenic v-myc/vraf expressing J2 retrovirus as previously described (95, 96) and differentiated into macrophages in media containing MCSF. Im-Macs were maintained in 10%FCS PSN DMEM (Thermo Fisher Scientific). Im-Macs lines have also been obtained from the BEI resources: TLR3, -4,- 7, -9, –2-9, and –2-4; MYD88; TRIF; TRAM; and TRIF-TRAM (BEI resources ATCC/NIAID; www.beiresources.org).

#### Primary BMDMs and BMDCs.

BMDMs and BMDCs were generated from the BM of 6- to 8-week-old female C57BL/6 mice. For BMDMs, cells were cultured in complete RPMI supplemented with 20 μg/mL of murine colony stimulating factor (M-CSF, Peprotech, 315-02); for BMDCs, cells were cultured in complete IMDM supplemented with 200 ng/mL of FMS-like tyrosine kinase 3 ligand (Flt3L, Peprotech, 250-31L). Cells were cultured for 10 days, with media exchange on day 5.

#### B16 F1 cell lines.

B16 F1 murine melanoma cell lines (ATCC, CRL-6326) and B16 F10 murine melanoma cell lines (ATCC, CRL-6475) were obtained from Miriam Merad (Precision Immunology Institute and Tisch Cancer Institute, Icahn School of Medicine at Mount Sinai, New York, New York, USA). Cells were maintained in complete RPMI media (as mentioned above). Cells were also IMPACT tested and were found free of contaminants and safe for in vivo injections into mice.

#### B16 F1 MMP-OE cell generation.

The multisite gateway cloning system was used for lentiviral plasmid assembly and to OE *Mmp2* and *Mmp9* with a lentiviral plasmid using the EF-1α promoter. *Mmp2* and *Mmp9* inserts were cloned into an entry plasmid. Gibson HiFi assembly was performed to clone the purified inserts into the entry vector (EF-1α ENTR A plasmid). Multisite gateway LR recombination was performed using Invitrogen’s Gateway LR Clonase II enzyme mix so that the inserts under the EF-1α promoter could be cloned into a destination vector containing puromycin resistance cassette (PuroR plasmid). Cells were cotransfected with *Mmp*-PuroR + GAG, VSV-G, and Rev plasmids; viral supernatants were concentrated by ultracentrifugation (71,900*g*, 2 hours, room temperature) and transduced into B16 melanoma cells.

#### B16 F1 Mmp2 CRISPR-KO generation.

Three sgRNAs for mouse *Mmp2* and a pLentiCas9-EGFP plasmid were obtained from Genscript. sgRNA 3 and 5 were selected for Lentiviral generation. HEK293T cells were transfected with plasmids plus Lentiviral packaging plasmids (Group-specific antigens [Gag] + Rev + Vesicular stomatitis virus G protein [VSV-G]), using Lipofectamine 3000. Viral supernatant was concentrated by ultracentrifugation (71,900*g*, 2 hours, room temperature). B16 F1 cells were transduced with pLentiCas9-EGFP and sorted based on GFP expression. Cas9-GFP^hi^ cells were retransduced with *Mmp2* sgRNA 3 or 5 lentivirus as described above. sgRNA expression selection was done by culture with puromycin. Mutation was confirmed with IDT’s Surveyor Mutation Detection Kit, and single cell clones were generated. The B16 F1 Cas9^hi^ sg3 D1 clone was selected experiments, herein known as F1 *Mmp2*-KO cells. F1 Cas9^hi^ no sgRNA were used as controls.

### Cell stimulation

Im-Macs, BMDCs, and BMDMs were seeded in 96 flat-bottom–well plates at 200,000 cells/well for primary cells and 100,000 cells/well for Im-Macs and stimulated with 5 μg/mL rhMMP2 (Enzo Life Sciences), rhMMP9 (MilliporeSigma), or vehicle control (Enzo Life Sciences); 100 ng/mL of Ultrapure LPS (Invivogen); Pam3CSK4, MALP2, and R848 (Invivogen); and 2 μg/mL HMW PolyI:C (Invivogen). Cells were stimulated for 16–20 hours for CBA and 8 hours for RT-PCR.

### Cytometric Bead Array (CBA)

Supernatant from stimulated cells was collected and frozen at –20°C until ready for use. CBA kits for mouse Inflammation (catalog 552364) and mouse Th1 and Th2 cytokines (catalog 551287) were purchased from BD Biosciences, and the procedure was adapted from the manufacturer’s directions. Analysis was done with the FCAP Software from BD Biosciences.

### Transient transfection

HEK293T cells (ATCC, CRL-3216, RRID: CVCL_0063) were plated in 10 cm plates and cultured overnight before transfection with 5 μg of each plasmid, using Lipofectamine 3000 at a 1:2 ratio DNA/lipofectamine in 1 mL of Opti-MEM reduced serum media for 16–20 hours.

### Western blotting

Cells were collected in lysis buffer, incubated on ice for 30 minutes, and centrifuged at 4°C at 16,000*g* for 10 minutes. Protein quantification was performed using the Bradford assay. Lysates were separated as described before (13). Proteins were resolved by SDS/PAGE and transferred to PVDF membranes. Membranes were blocked, probed overnight with primary antibodies, and incubated with secondary antibodies for 2 hours at room temperature. A complete list of primary and secondary antibodies used is in Supplemental Table 1.

### Plasmid design and preparation

All plasmid constructs were designed in house and generated by Genscript (www.genscript.com). *Mmp2*–Flag-tagged and all MMP2 domains plus Flag tags were generated based on codon-optimized full-length murine *Mmp2* sequence. Murine *Tlr2*-HA and murine *Tlr4*-Myc constructs were also codon optimized. The *Mmp2*-Pex (PEX-LV) construct was a gift from Inder Verma (Addgene plasmid 12120) (97).

### Co-IP

A total of 200 μg of protein lysate was used for co-IP. Protein was incubated with anti-FLAG M2 magnetic beads (Sigma-Aldrich), monoclonal anti-HA agarose antibody (Roche), or anti–c-MYC Agarose Affinity gel antibody (Roche) overnight. For immunoprecipitation, samples were eluted using 3× FLAG peptide, HA peptides, or c-MYC peptide. Samples were further analyzed using Western blotting.

### BM chimeras

#### BM isolation.

Tibia and femurs of donor mice were collected. Bone tips were cut and BM flushed using a 27 G needle and RPMI into 50 mL tubes with a 70 μm strainer. Cells were centrifuged (300*g*, 7 minutes, 4°C) and incubated with ACK lysis buffer for RBC lysis.

#### Irradiation and BM injections.

Mice were irradiated with 2 doses of 600 rads (6 Gy), 4 hours apart. A total of 100 μL (4 *×* 10^6^ cells) of cell suspension was injected into the tail vein. Six to 8 weeks after BM transfers, blood was collected, lysed of RBCs, and stained with an antibody cocktail mix for 30 minutes. FACS was performed to check CD45.1 versus CD45.2 engraftment.

### Tumor processing

Tumors were collected between days 15 and 21 after injections and dissociated using the mouse tumor dissociation kit (catalog 130-096-730) and gentleMACS from Miltenyi Biotec, using program 37°C_m_TDK1. Macerated tumors were passed through a 70 μm cell strainer, washed with RPMI, and centrifuged at 300*g* for 8 minutes at 4°C. Cell pellets were resuspended in 2 mL of ACK lysis buffer for 2 minutes, at room temperature, and washed in 1× PBS (300g, 8 minutes, 4°C). Single-cell suspension was analyzed by FACS or CyTOF.

### FACS analysis of TILs

Single-cell suspensions were aliquoted into round-bottom 96-well plates, resuspended in 100–150 μL of antibody mixes, and incubated at 4°C, for 30 minutes, in the dark. For intracellular staining (ICS), samples were processed with eBioscience FoxP3 Staining Kit (as per manufacturer’s instructions) and incubated in 150 μL of ICS Ab mix at 4°C for 30 minutes. Antibodies used are listed in Supplemental Table 2.

### CyTOF

In total, 3 *×* 10^6^ to 5 *×* 10^6^ live cells were taken for CyTOF at the Human Immune Monitoring Core facility of the Tisch Cancer Institute, Icahn School of Medicine at Mount Sinai (https://icahn.mssm.edu/research/human-immune-monitoring-center), where they were prepared for CyTOF as per the facility protocol. One lymphoid and 1 myeloid panel were designed, and samples were analyzed based on these. Antibodies panel and metal conjugations were generated and optimized by the Human Immune Monitoring Core facility. Supplemental Table 3 contains the complete list of metal-conjugated antibodies.

### CyTOF analysis

Analysis was performed using R software. Barcoded FCS files were read and transformed using a hyperbolic inverse sine (asinh) with a cofactor of 5 using R/Bioconductor/flowCore 1.48.1. Cell counts were between 196,000 and 558,000. Unsupervised multidimensional scaling (MDS) plots were generated using R/Bioconductor/limma 3.38.3 (98). Hierarchical clustering with Euclidean distances was performed using R/Bioconductor/FlowSOM 1.14.1 and R/Bioconductor/ConsensusClusterPlus with a maximum of 25 clusters. tSNE was calculated using R/CRAN/Rtsne 0.15. Statistical analysis was performed using binomial generalized linear mixed-effects model (GLMM) from the R/CRAN/lme4 1.1-20 package. *P* values were adjusted using FDR. Heatmaps were generated with R/CRAN/pheatmap 1.0.12, and all other plots were generated using R/CRAN/ggplot2 3.1.0.

### B16 tumors fixation, freezing, and sectioning

B16 melanomas were collected and incubated in periodate-lysin-paraformaldehyde (PLP) solution overnight at 4°C. Tissues were then washed in a sodium phosphate buffer (mixture of sodium phosphate monobasic and dibasic buffers) for 1–3 minutes and dehydrated by successive sucrose gradients (10%, 20%, and 30%), each for 2 hours at 4°C. They were then embedded in OCT, frozen, and kept in –80°C. Using a cryostat, 7–10 μm sections were cut and transferred into superfrost slides and kept at –20°C.

### Immunofluorescence of frozen sections

Frozen slides were permeabilized with 1× TBS + 0.1% Triton-X for 15 minutes at room temperature; they were then washed with 1× TBS. Tissues were blocked with 10% BSA/TBS for 15–20 minutes at room temperature, and slides were incubated with primary antibodies overnight at 4°C. Slides were washed for 10 minutes in TBS at room temperature and incubated with secondary antibodies at room temperature in the dark for 2 hours. Slides were washed twice in 1× TBS and then mounted using ProLong Antifade Reagent with DAPI (Thermo Fisher Scientific, P36931). Supplemental Table 4 includes a list of antibodies used.

### Statistics

For all graphical analyses, mean ± SEM values were displayed. Student’s *t* test (2-tailed, unpaired), 1-way or 2-way ANOVA with Dunnett’s post hoc test, or multiple 2-tailed, unpaired *t* test with Holm-Sidak correction for multiple comparisons were calculated using Prism 8 (GraphPad). A *P* value less than 0.05 was considered significant.

### Study approval

All experimental procedures using mice were approved by the IACUC of the Icahn School of Medicine at Mount Sinai and were conducted in accordance with institutionally approved protocols and guidelines for animal care and use.

## Author contributions

LRMB designed and conducted the experiments, conducted data analysis, and drafted the manuscript. CBM assisted with tumor collection and processing experiments and generated some of the lentiviruses used in the B16 transduction experiments. GB performed CyTOF and RNA-seq analysis. LRMB, MS, and NB interpreted and discussed the data. MM provided some mouse lines and gave insight on experiment planning and data discussion. NB supervised experiment designs, developed the manuscript, and secured funding for the project.

## Supplementary Material

Supplemental data

## Version 1 05/25/2021

In-Press Preview

## Version 2 06/22/2021

Electronic publication
