# Supplementary material for: MMP2 and TLRs modulate immune responses in the tumor microenvironment
Source: JCI Insight. 2021 Jun 22;6(12):e144913. doi: 10.1172/jci.insight.144913 (PMC8262464; doi:10.1172/jci.insight.144913)
Supplement: Supplemental data [file jciinsight-6-144913-s193.pdf]

SUPPLEMENTAL DATA

Supplemental Figures

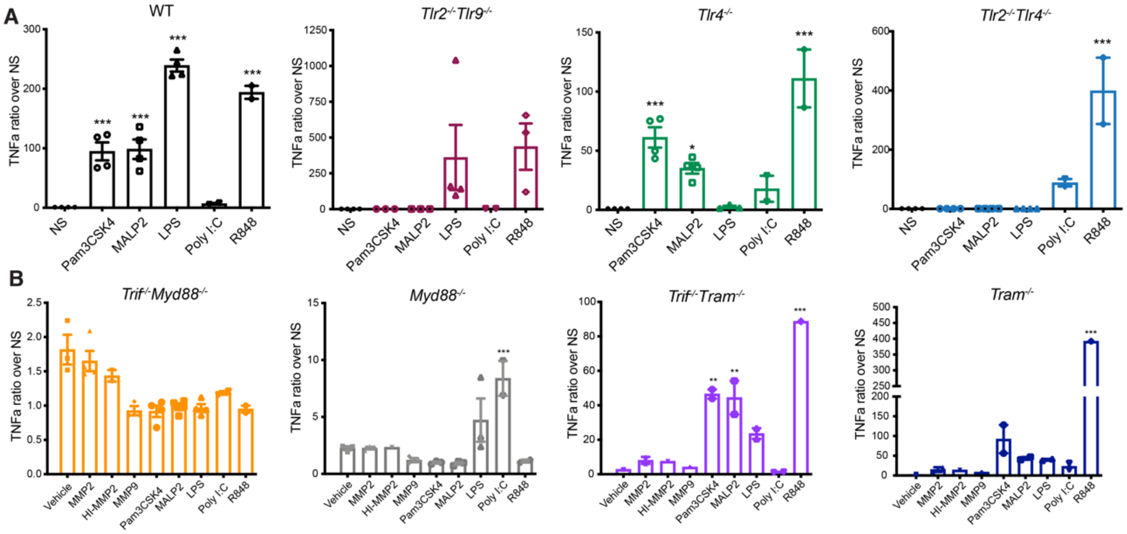

Figure S1. MMP2 and TLR agonist responses in immortalized BM cells: Pro-inflammatory cytokine secretion from immortalized BMDMs stimulated with MMP2, HI MMP-2, MMP9, Vehicle and TLR agonists controls *in vitro*. TNFα from WT and *Tlr*-deficient Im-Macs (A) and from TLR-signaling molecules (B) in response to stimulation. Each graph represents the ratio over unstimulated from each cell line depicted. Stimulations were performed with $2x10^5$ cells per condition in 200µl volume. 16-18 hours post stimulation, supernatants were collected and cytometric bead array for mouse inflammatory cytokines was performed. Data is representative of 2-4 experiments with mean ± SEM. * is p<0.05 and ** is p<0.01 and *** is p<0.001. One-way ANOVA with Dunnet's post hoc comparison.

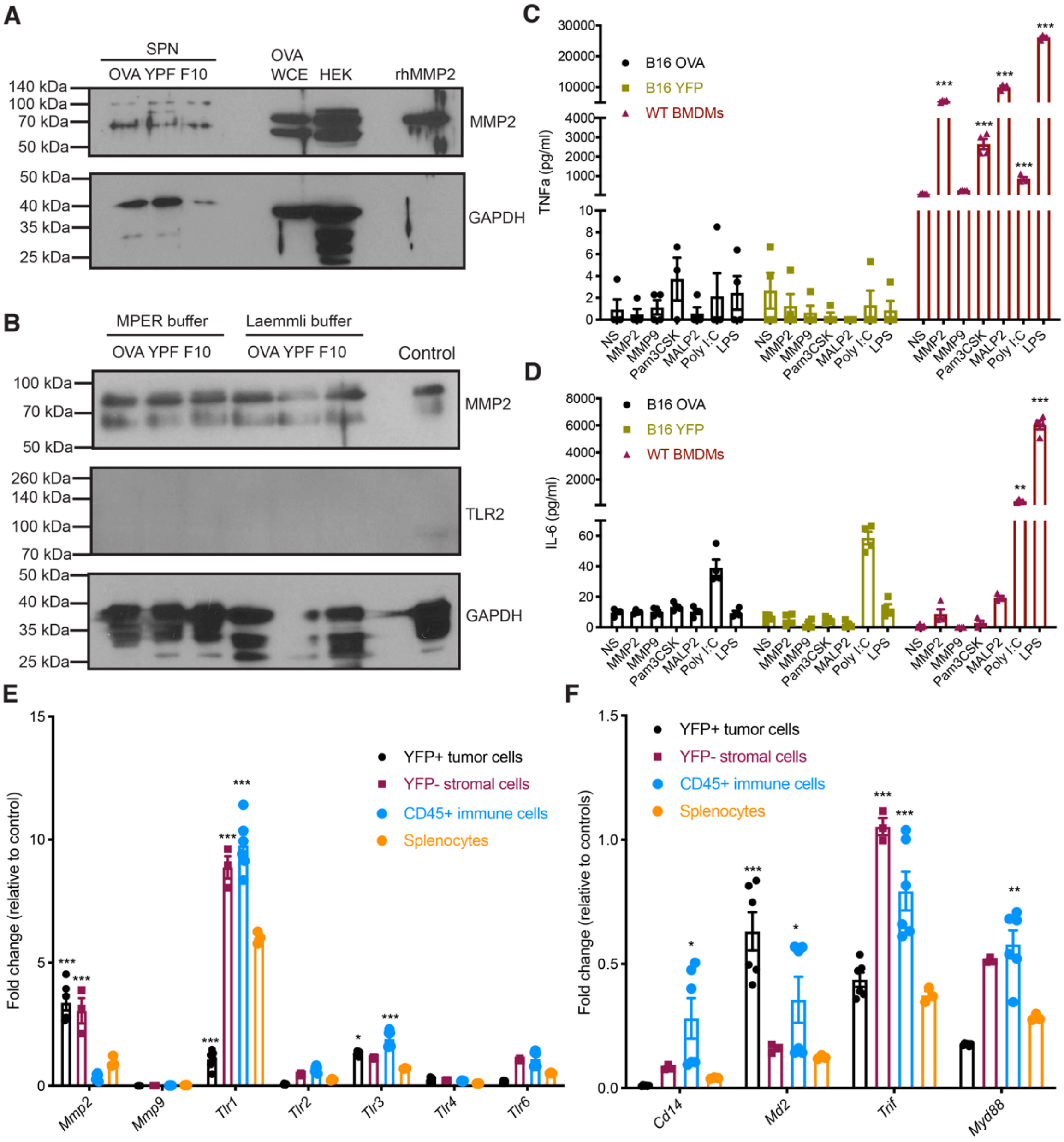

Figure S2. MMP2 and TLR expression in B16 melanoma cells: A-B. Western Blot analysis of MMP2 and TLR2 in B16 F1 and F10 cell lines from supernatant (SPN; A) and whole cell extract (WCE; B). C-D. TNFα (C) and IL-6 (D) secretion by B16 F1 cells after in vitro activation. Cells were activated with different TLR agonists for 20 hours and supernatant was collected and analyzed by CBA. Data is representative of 3 experiments with mean ± SEM. * $p > 0.05$, ** $p > 0.01$ and *** $p > 0.001$. Two-way ANOVA with Dunnett's post hoc correction for multiple

comparisons (significance against BMDMs). E-F. MMPs and TLR (E) and TLR-adaptors (F) mRNA gene expression comparing sorted tumor and stromal cells to whole tumor. Fold change was normalized to *Gapdh*. Each sample was done in triplicates and data is representative of 2 experiments with mean ± SEM. * $p > 0.05$, ** $p > 0.01$, *** $p > 0.001$. Two-way ANOVA with Dunnett's post hoc correction for multiple comparisons (significance against splenocytes).

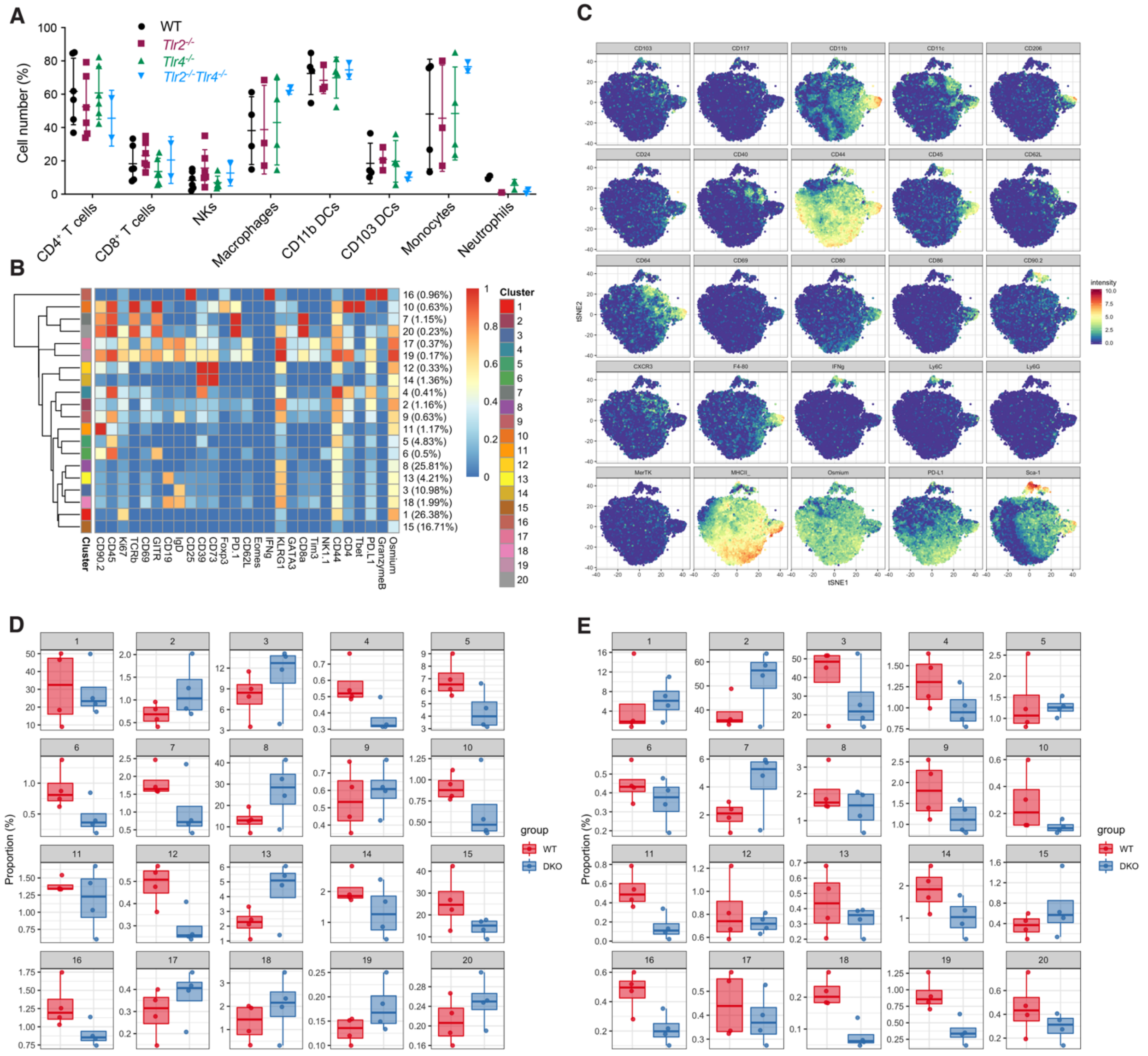

Figure S3. Differences in the tumor microenvironment in *Tlr2*$^{-/-}$*Tlr4*$^{-/-}$ mice: 3x10^{5} B16 F1 cells were injected sub-cutaneously into WT or TLR deficient mice and tumors were analyzed 15-19 days later. A. FACS analysis of immune cells in WT, *Tlr2*$^{-/-}$, *Tlr4*$^{-/-}$ and *Tlr2*$^{-/-}$*Tlr4*$^{-/-}$ mice at day 15. Data is representative of 3 experiments with mean ± SEM. Two-way ANOVA with Dunnett's post hoc correction for multiple comparisons. B-E. CyTOF analysis of tumors between WT and *Tlr2*$^{-/-}$*Tlr4*$^{-/-}$ mice. Heatmap of the 20 identified clusters from the CyTOF panels showing the expression of antigens for each cluster (B) with plots of single antigens expressed in the tumors at day 19

(C). Lymphoid clusters (D) and myeloid clusters (E) of the differentially expressed between WT and *Tlr2*$^{-/-}$*Tlr4*$^{-/-}$ mice. Data is representative of 3-5 mice with mean ± SEM. * is $p<0.05$, ** is $p<0.01$. p values were adjusted using FDR. Statistical analysis was performed using binomial generalized linear mixed-effects model (GLMM).

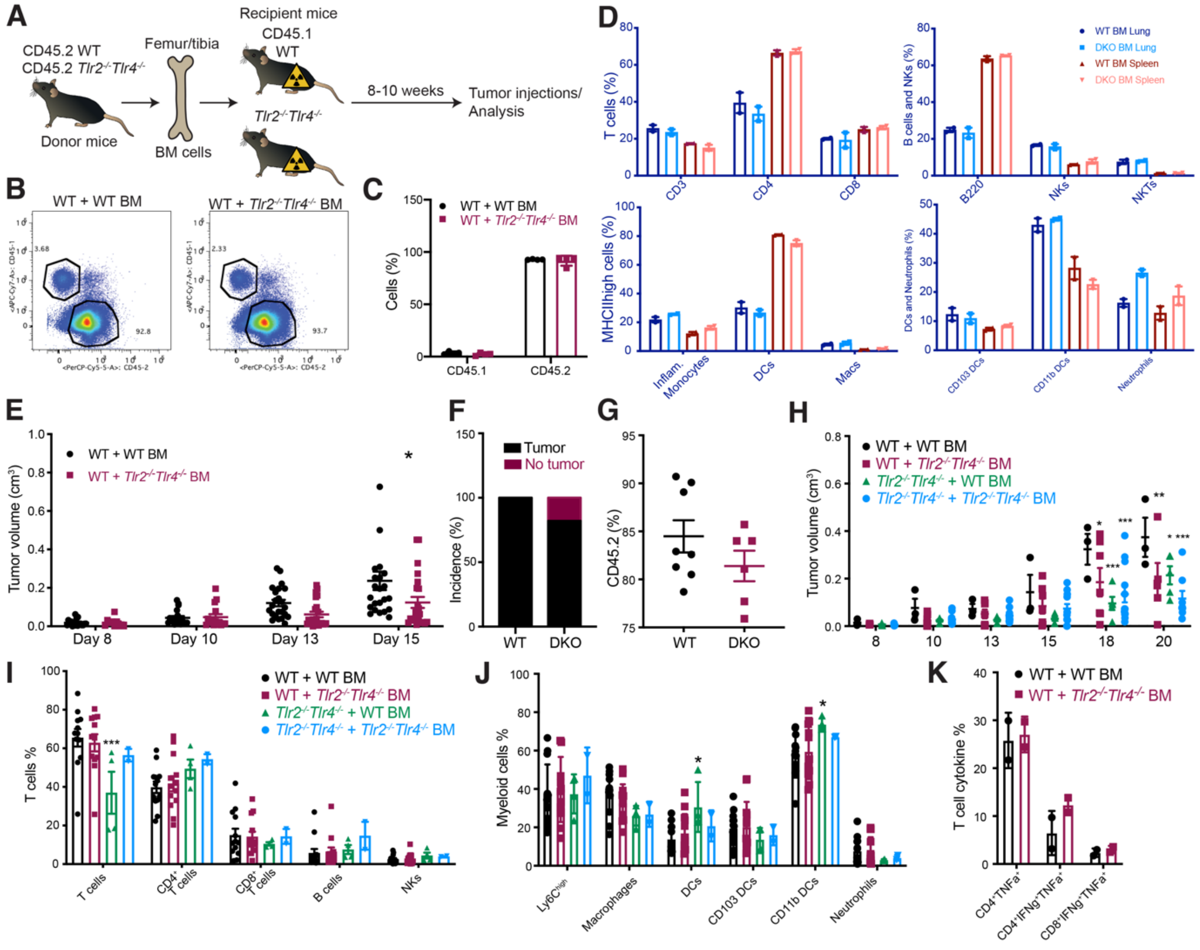

Figure S4. TLR2 and TLR4 expression is required in the hematopoietic compartment to support tumor growth: CD45.1 WT mice were irradiated with 1200 Grays (2x 600 Grays, 3 hours apart) and injected with $4x10^6$ WT or *Tlr2*$^{-/-}$*Tlr4*$^{-/-}$ (DKO) BMs. A. BM transfer schematic. B-C. CD45.1 versus CD45.2 engraftment in blood WT mice that received WT and *Tlr2*$^{-/-}$*Tlr4*$^{-/-}$ BM cells, 6-8 weeks post transfer. Representative plots (B) and cell percentage values (C) are displayed. D. Engraftment of cells in lungs and spleen of mice, 9-10 weeks post transfer. Reconstitution was similar in mice that received WT (dark colors) or DKO (lighter colors) BMs. N=10 mice per group. Mean ± SEM. Two-way ANOVA with Dunnett's post hoc correction for multiple comparisons. E-G. Mice were subcutaneously injected with $3x10^5$ B16 F1 cells and tumors were measured up to 16 days. Tumor volume (E), incidence (F) and total hematopoietic cell number in tumor infiltrates

(G) was analyzed. N=23 mice per group for tumor growth. Mean ± SEM. * is $p<0.05$ **is $p<0.01$ and ***is $p<0.001$. Two-way ANOVA with Sidak's post hoc correction for multiple comparisons. H. Tumor growth comparison including *Tlr2*$^{-/-}$*Tlr4*$^{-/-}$ recipients. Data is representative of 3 experiments. I-K. Immune cell infiltrates in tumors from WT and *Tlr2*$^{-/-}$*Tlr4*$^{-/-}$ mice that received WT or *Tlr2*$^{-/-}$*Tlr4*$^{-/-}$ BM cells. Lymphoid (I), Myeloid (J) and T cell cytokine (K) infiltrates between the groups. Data is representative of 3 experiments. For H-J: Mean ± SEM. * is $p<0.05$ **is $p<0.01$ and ***is $p<0.001$. Two-way ANOVA with Dunnett's post hoc correction for multiple comparisons.

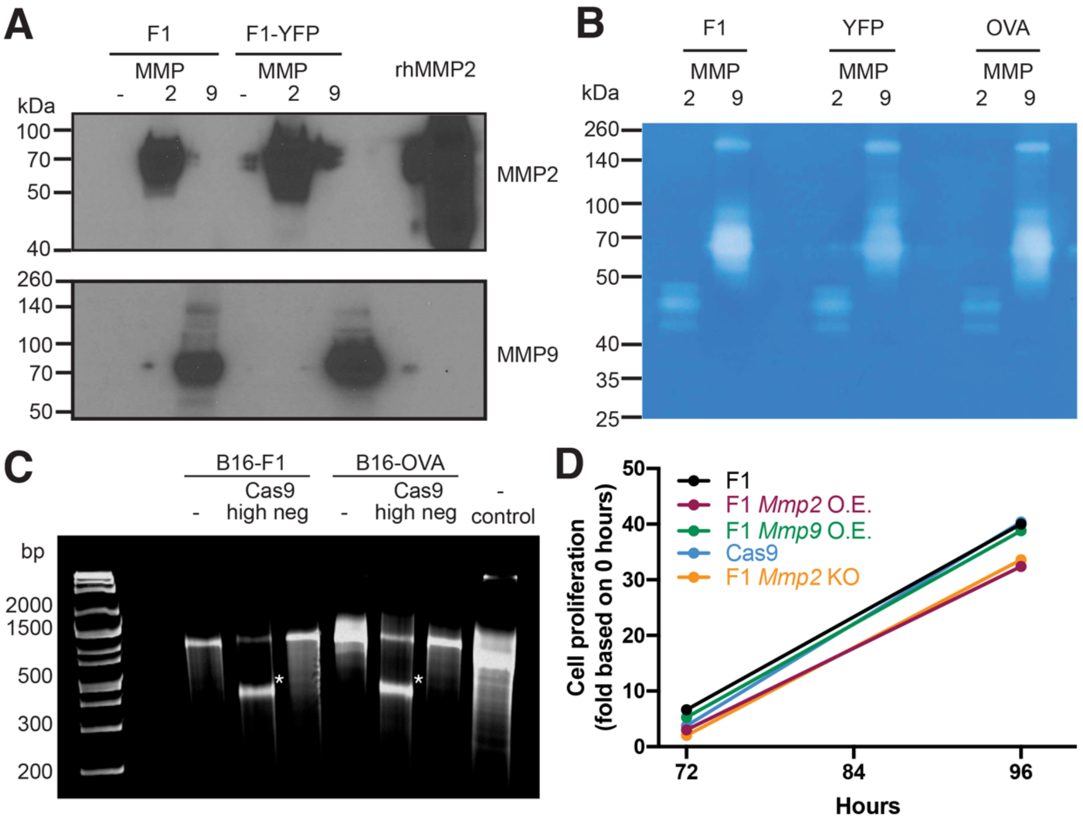

Figure S5. MMP-2 overexpression and depletion in B16 cells: The levels of MMP-2 in the B16 was modulated by overexpression and knock out CRISPR systems. A. Western blot images confirm overexpression of MMP-2 (~72kDa) and MMP-9 (~90kDa) in the B16 cells. B. Zymography assay confirms secretion of active MMP-2 and MMP-9 in the B16 cell lines. C. Surveyor assay of B16 F1 and OVA lines that were KO for MMP2 using a CRISPR-Cas9-GFP system. * indicate the bands that show effective endonuclease activity and mismatch. D. Cell growth rates between the different cell lines (MMP-2 competent and deficient cells).

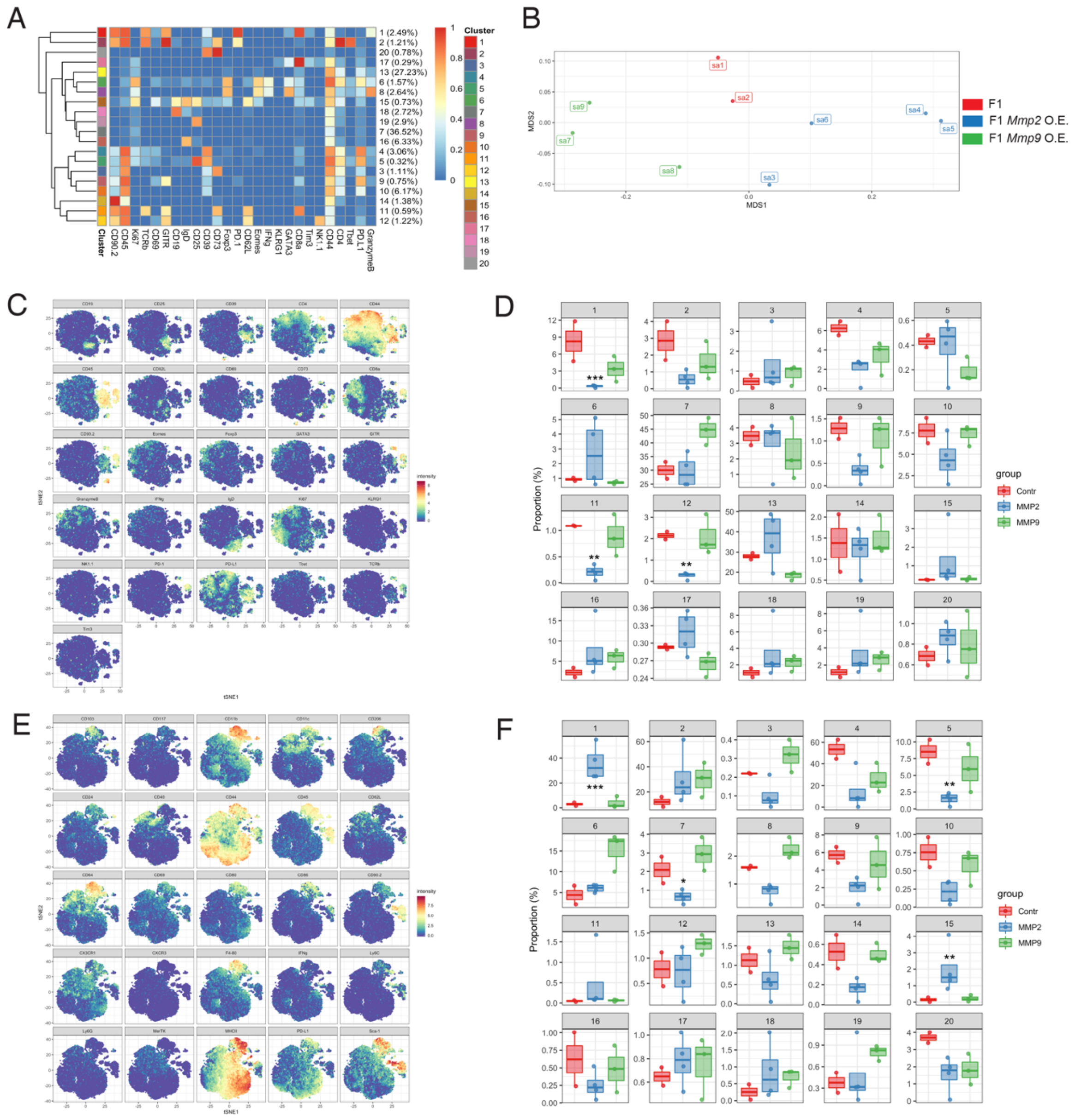

Figure S6. CyTOF comparison of tumors with MMP overexpression: CyTOF analysis of WT mice injected with F1, F1 MMP-2 OE and F1 MMP-9 OE tumors. A. Heatmap plot of the 20 clusters identified by CyTOF. B. Multidimensional scaling plot showing samples distribution/grouping. C. viSNE plot of single antigen expression in lymphoid panel. D. Differential expression of lymphoid clusters. E. viSNE plot of single antigen expression in myeloid panel. F. Differential expression of myeloid clusters. Data is representative of 3-5 mice with mean ± SEM. * is p<0.05, ** is p<0.01.

p values were adjusted using FDR. Statistical analysis was performed using binomial generalized linear mixed-effects model (GLMM).

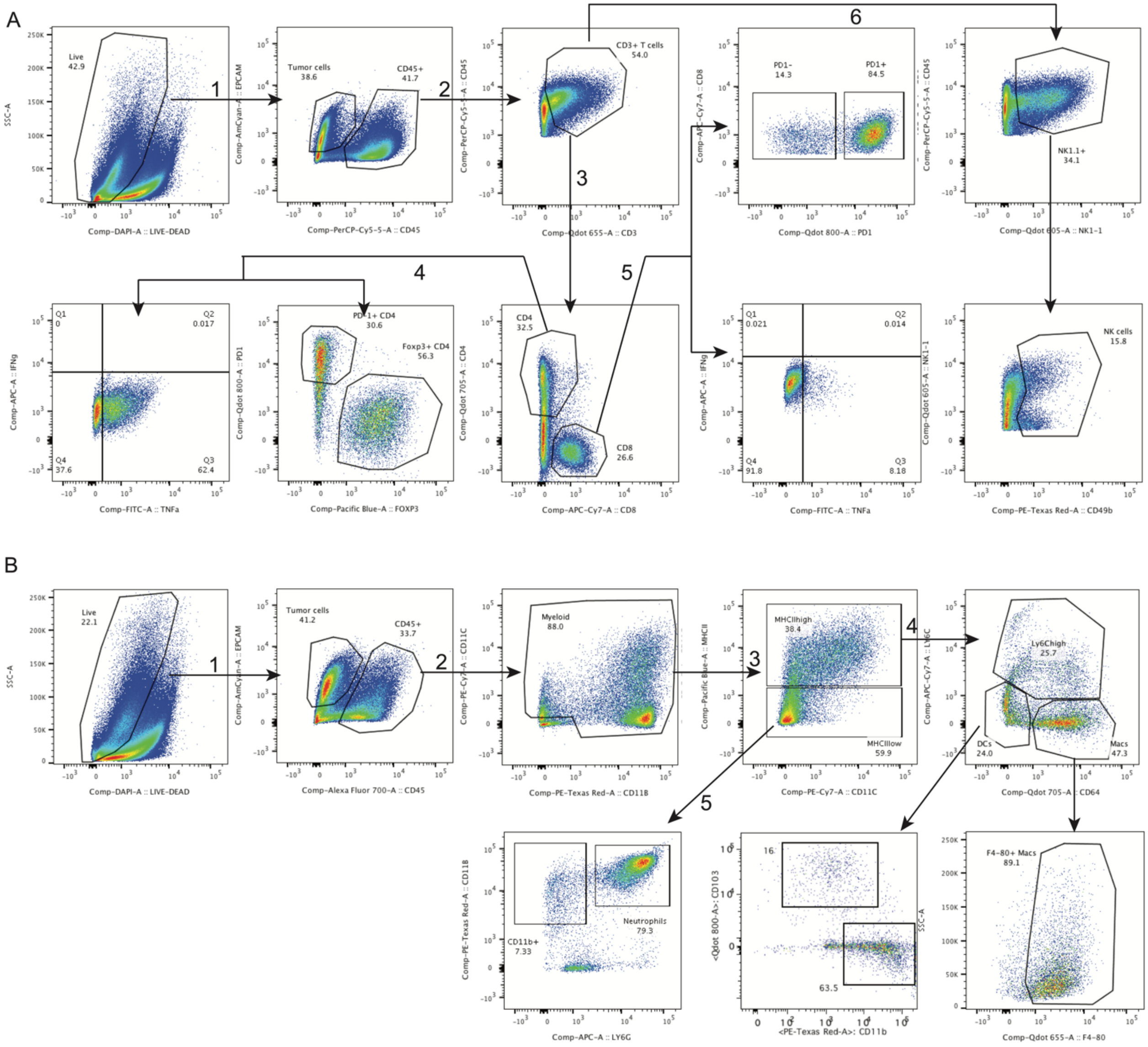

Figure S7. FACS gating strategy of *Mmp2* KO and WT B16 tumors at day 19: Lymphoid panel gating strategy is shown (A). Cells were gated on Live (1), CD45^{+} hematopoietic cells (2), then CD3^{+} T cells (3), separated on CD4 and CD8 T cells. Within the CD4 T cells, TNFα, IFNγ, PD-1 and FoxP3 populations were derived (4). From the CD8 T cells, TNFα, IFNγ and PD-1 populations were derived. B. In the myeloid panel, cells were gated on Live (1), CD45^{+} hematopoietic cells (2), then myeloid, as CD11c^{+} CD11b^{+} cells (3). From the myeloid cells, MHCII$^{high\ vs\ low}$ were separated (based on MHCII vs CD11c expression). From the MHCII^{high} cells, DCs (Ly6C^{-}, CD64^{-}

), Macrophages (Ly6C^{-}, CD64^{+}) and Ly6C^{high} cells are gated (4). DCs were further gated into CD103^{+} and CD11b^{+} subpopulations and from the macrophages, F4-80^{+} subpopulations. From the MHCII^{low} cells, Neutrophils were gated (CD11b^{+}, Ly6G^{+}; 5). Data is representative of 3 independent experiments with mean ± SEM. N=8-10 mice per group.

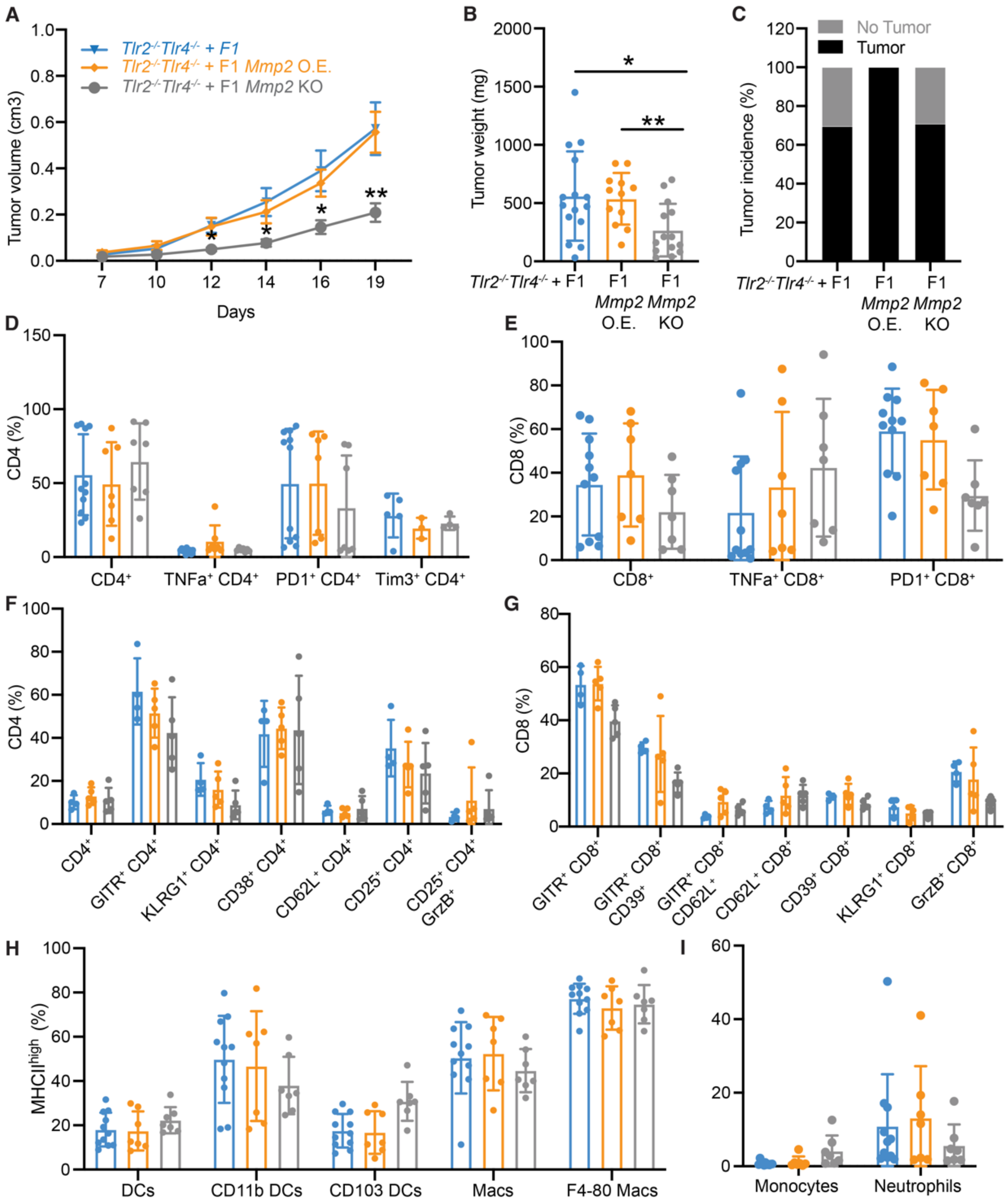

Figure S8. Lack of host TLR2 and TLR4 impacts the effects MMP2 expression in tumors: A-C. Tumor development in *Tlr2*$^{-/-}$*Tlr4*$^{-/-}$ mice injected with B16 F1, F1 *Mmp2* O.E. and F1 *Mmp2* KO cells. Tumor growth kinetics (A), tumor weight at collection day 19 (B) and tumor incidence (C). Mean ± SEM. * is $p<0.05$ **is $p<0.01$ and ***is $p<0.001$. Two-way ANOVA with Dunnett's post hoc correction for multiple comparisons. D-I. FACS comparison of immune infiltrates in *Tlr2*$^{-/-}$*Tlr4*$^{-}$

$^{/-}$ mice. CD4 (D, F), CD8 (E, G) and Myeloid (H-I) populations percentages. Data is representative of 3 experiments with N = 6-10 mice per group with mean ± SEM. * is $p<0.05$ and ** is $p<0.01$ and *** is $p<0.001$. Two-way ANOVA with Dunnett's post hoc correction for multiple comparisons.

Supplemental Tables

Table S1: List of antibodies used for Western Blot

Antibody	Host	Manufacturer	Catalog number
Monoclonal ANTI-FLAG® M2 antibody	Mouse	Sigma-Aldrich	F1804
Anti-HA (12CA5)	Mouse	Roche	11 666 606 001
Anti-c-myc	Mouse	Roche	11 667 149 001
Toll-like Receptor 4 (D8L5W)	Rabbit	Cell Signaling	14358
Toll-like Receptor 2 (E1J2W)	Rabbit	Cell Signaling	13744
Anti-MMP2 antibody	Rabbit	Abcam	ab37150
MMP2 (H76)	Rabbit	Santa Cruz Biotechnology	Sc-10736
Anti-mouse IgG, HRP-linked antibody	Horse	Cell Signaling	7076
Anti-rabbit IgG, HRP-linked Antibody	Goat	Cell Signaling	7074

Table S2: List of antibodies used for flow cytometry

Target	Fluorochrome	Manufacturer	Catalog number	Target	Fluorochrome	Manufacturer	Catalog number
Epcam	BV510	BioLegend	118231	CD44	APC-Cy7	BioLegend	103027
CD45	APC-Cy7	BioLegend	103116	PD-L1	BV711	BioLegend	124319
CD3	BV650	BioLegend	100229	PD-1	BV785	BioLegend	135225
CD4	BV711	BioLegend	100447	CD31	PECy7	BioLegend	102417
CD8	A700	BioLegend	100729	CD47	PE-Dazzle 594	BioLegend	27521
B220	PercPCy5.5	BioLegend	103236	OX40L	APC	BioLegend	108811
NK1.1	BV605	BioLegend	108739	Ki67	BV605	BioLegend	652413
CD49b	PE-CF594	BD Biosciences	562453	XCR1	APC	BioLegend	148205
IFNγ	APC	BioLegend	505810	Sirpα	APC-Cy7	BioLegend	144017
TNFα	FITC	eBiosciences	11-7321-82	Siglec-H	PE	eBiosciences	12-0333-80
FoxP3	BV421	BioLegend	126419	CD80	PECy5	eBiosciences	15-0801-81
CD11c	PECy7	BioLegend	117318	CD86	BV650	BioLegend	105035
CD11b	PE-CF594	BD Biosciences	562287	CD206	A700	BioLegend	141733
MHCII	e450	eBiosciences	14-5321-82	CD115	BV605	BioLegend	104526
CCR2	PE	BioLegend	150609	CX3CR1	BV785	BioLegend	149029
Ly6C	APC-Cy7	BioLegend	128026	CD24	BV421	BioLegend	101825
Ly6G	APC	BioLegend	127614	CD69	APCCy7	BioLegend	104526

CD64	BV711	BioLegend	139311	CD62L	PE-Dazzle 594	BioLegend	104448
CD103	BV786	BD Biosciences	564322	CD25	BV650	BioLegend	102038
F4-80	BV650	BioLegend	123149	Tim3	APC	eBiosciences	17-5871-80
Sca1	BV421	BioLegend	08127	Lag3	PercPCy5.5	BioLegend	125212
gp38	PE	BioLegend	127407	CTLA-4	PE	BioLegend	106305
GATA3	PE-CF594	BD Biosciences	563510	T-bet	BV412	BD Biosciences	563318
IL-4	PE-Cy7	BioLegend	504117	IL-13	PE-Cy7	eBiosciences	25-7133-80
FoxP3	PE	BioLegend	126403	Live-Dead	Blue	Invitrogen	L23105

Table S3: List of antibodies used for CyTOF

Channel	Lymphoid Panel Markers	Channel	Myeloid Panel Markers
**113 In**	CD90.2	113 In	CD90.2
**115 In**	C45	115 In	CD45
**141 Pr**	Ki67	141 Pr	Ly6G
**143 Nd**	TCRb	142 Nd	CD11c
**145 Nd**	CD69	144 Nd	CD24
**147 Sm**	CD375/GITR	145 Nd	CD69
**149 Sm**	CD19	146 Nd	F4-80
**150 Nd**	IgD	148 Nd	CD11b
**151 Eu**	CD25	152 Sm	CD86
**154 Sm**	CD39	156 Gd	CD64
**155 Gd**	CD73	158 Gd	CD117
**158 Gd**	Foxp3	159 Tb	CD40
**159 Tb**	PD-1	160 Gd	CD62L
**160 Gd**	CD62L	161 Dy	CD103
**164 Dy**	Eomes	162 Dy	Ly6C
**165 Ho**	IFNg	164 Dy	Sca-1
**166 Er**	KLRG1	165 Ho	IFNg
**168 Er**	CD8a	167 Er	CXCR3
**169 Tm**	Tim3	168 ER	MerTK
**170 Er**	NK1.1	169 Tm	CD206
**171 Yb**	CD44	171 Yb	CD44
**172 Yb**	CD4	173 Yb	CD80
**173 Yb**	Tbet	174 Yb	MHCII (I-A/I-E)
**175 Lu**	PD-L1	175 Lu	PD-L1
**176 Yb**	Granzyme-B	176 Yb	CX3CR1

Table S4: List of antibodies used for immunofluorescence

Target	Host	Fluorochrome	Manufacturer	Catalog number	Application
Granzyme B	Goat	Unconjugated	R&D Systems	AF1865	IF, primary
NK1.1	Mouse	Unconjugated	BioLegend	108701	IF, primary
CD8α	Rat	Unconjugated	BioLegend	100702	IF, primary
CD3e	Rabbit	Unconjugated	Abcam	ab5690	IF, primary
Ki67	Rat	Unconjugated	BioLegend,	151202	IF, primary
Anti-Rat IgG (H+L)	Goat	Alexa Fluor 488	Thermo Fisher Scientific	A11006	IF, secondary
Anti-Rat IgG (H+L)	Goat	Alexa Fluor 594	Thermo Fisher Scientific	A11007	IF, secondary
Anti-Rabbit IgG (H+L)	Goat	Alexa Fluor 488	Thermo Fisher Scientific	A32731	IF, secondary
Anti-Rabbit IgG (H+L)	Goat	Alexa Fluor 594	Thermo Fisher Scientific	A32740	IF, secondary
Anti-Mouse IgG (H+L)	Goat	Alexa Fluor 488	Thermo Fisher Scientific	A32723	IF, secondary
Anti-Mouse IgG (H+L)	Goat	Alexa Fluor 594	Thermo Fisher Scientific	A32742	IF, secondary
Anti-Hamster IgG (H+L)	Goat	Alexa Fluor 488	Thermo Fisher Scientific	A21110	IF, secondary
Anti-Hamster IgG (H+L)	Goat	Alexa Fluor 594	Thermo Fisher Scientific	A21113	IF, secondary
Anti-Goat IgG (H+L)	Donkey	Alexa Fluor 488	Thermo Fisher Scientific	A32814	IF, secondary

Experimental Methods

Cell lines and cell culture

Primary BMDMs and BMDCs: BMDMs and BMDCs were generated from the bone marrow of 6–8-week-old female C57BL/6 mice. For the generation of BMDMs cells were cultured in RPMI with 10% FCS, 10mM Hepes, Pen/Strep, 1mM Sodium Pyruvate, 1x β-Mercaptoethanol (all from Gibco) supplemented with 20ug/ml of murine colony stimulating factor (M-CSF, Prepotech #315-02). To generate BMDCs, bone marrow cells were and cultured in IMDM (Gibco) with 10% FCS (Gibco), Pen/Strep, 1mM Sodium Pyruvate (Gibco), 10mM Hepes and 1x β-Mercaptoethanol (Gibco) supplemented with 200ng/ml of FMS-like tyrosine kinase 3 ligand (Flt3L, Prepotech #250-31L). In both protocols, 1-2x10^6 cells/ml were cultured in 10-cm plates for 10 days, with media exchange on day 5.

B16 F1 MMP-overexpressing cell generation: we used the multisite gateway cloning system for lentiviral plasmid assembly and to overexpress *Mmp2* and *Mmp9* with a lentiviral plasmid using the EF-1α promoter. *Mmp2* and *Mmp9* inserts were cloned into an entry plasmid. Inserts were amplified by polymerase chain reaction (PCR) using the following primers: Fwd *Mmp2* EF1a ENTR (TAAGCTTGGTACCGAGCTCGGCCACCATGGAGGCTAGG), Rev *Mmp2* EF1a ENTR (ACTGTGCTGGATATCTGCAGTCAGCAGCCCAGCCAATC), Fwd *Mmp9* EF1a ENTR (TAAGCTTGGTACCGAGCTCGGCCACCATGAGTCCCTGG) and Rev *Mmp9* EF1a ENTR (ACTGTGCTGGATATCTGCAGTCAAGGGCACTGCAGGAG). Amplified inserts were resolved on agarose gel and gel amplified using Qiagen's Qiaquick gel extraction kit (#28704). Gibson HiFi assembly was performed to clone the purified inserts into the entry vector (EF-1α ENTR A plasmid) and then constructs were transformed into DH5α competent cells (Thermo Fisher Scientific, #18265017). Picked colonies were isolated and cultured for miniprep for DNA isolation (Qiagen, #27104). Colony PCR was performed using the following primers: *Mmp2* EF1a ENTR

Fwd colony (GTACCGAGCTCGGCCACCATGGAGGCT), *Mmp9* EF1a ENTR Fwd colony (GGTACCGAGCTCGGCCACCATGAGTCCC) and pEF1a ENTR Rev colony (GGTGATGGTGATGATGACCGGTACGCGTAG). A few of the selected colonies picked from the PCR were sequenced and then one colony each for *Mmp2* and *Mmp9* was chosen. Multisite gateway LR recombination was performed using Invitrogen's Gateway LR Clonase II enzyme mix so that the inserts under the EF-1α promoter could be cloned into a destination vector containing puromycin resistance cassette (PuroR plasmid). Cloned products were transformed into Stlb3 competent cells (Thermo Fisher Scientific, #C737303), colonies were picked and grown for miniprep isolation and diagnostic digestion using restriction enzymes (R.E.). Selected colonies were then grown into large cultures and isolated by endotoxin-free maxiprep (Qiagen, #12362). Finally, purified plasmids were co-transfected with GAG, VSV-G and Rev plasmids into HEK293T cells using lipofectamine 3000 (Thermo Fisher Scientific, #L3000015) or Calcium Chloride ($CaCl_2$) and viral supernatants were collected and concentrated by ultra-centrifugation (20000 g, 90 min). Concentrated viruses were transduced into B16 melanoma cells and transduced cells were selected using puromycin.

B16 F1 Mmp2 CRISPR knock out generation: sgRNAs for mouse *Mmp2* were ordered from Genscript (www.genscript.com), in addition to pLentiCas9-EGFP plasmid. A total of three sgRNA were ordered – sgRNA #3 (Sequence: GATGTGGCCAACTACAACTT; Position: Exon 2; targets the negative strand), sgRNA #4 (Sequence: TGCGGAAGCCAAGATGTGGC; Position: exon 2; targets the negative strand) and Sequence: CATTGGTTACACACCTGACC; Position: Exon 3; targets the positive strand). Plasmids were grown and isolated by maxiprep using Qiagen's Endo-free maxiprep kit (#12362). sgRNA #3 and #5 were then selected for Lentiviral generation. HEK293T cells were transfected with pLentiCas9-EGFP plasmid or sgRNA #3 or #5 plasmids + Lentiviral packaging plasmids (Gag+Rev+VSV-G), using Lipofectamine 3000 (Thermo Fischer Scientific # L3000015) in a 1:2 ration DNA:LP3000. 48 hours post transfection, viral supernatants

were collected, and virus was concentrated by ultra-centrifugation at 20000rpm, for 90 minutes at 4°C, then resuspended in 1x PBS and frozen. B16 cells were cultured at 1.5 x 10^5 cells per well in 6-well plates and transduced with pLentiCas9-EGFP concentrated virus at 1:1000 and 1:2000 dilution in the presence of polybrene at 4µg/ml concentration. Cells were spun at 2000 rpm for 20 min at RT and then cultured for 2-3 days. Confluent cells were then sorted based on their GFP expression, which indicated Cas9 expression. Cas9-GFP^{high} cells were then re-plated on 6-well plates in a 1.5x10^5 cells per well concentration and re-transduced with *Mmp2* sgRNA #3 or #5 lentivirus at a 1:1000 and 1:2000 dilution as described above. 2-3 days post *Mmp2* sgRNA lentivirus transduction, cells were cultured in the presence of puromycin (1 µg/ml and 2.5 µg/ml) for sgRNA expression selection. gDNA was isolated from puromycin resistant cells for confirming mutation using IDT's Surveyor Mutation Detection Kit (#706020). Finally, after confirming Cas9-*Mmp2* mutation had occurred, we generated single cell clones for a more homogenous population of B16 cells that are KO for *Mmp2*, selecting the B16 F1 Cas9^{high} sg3 D1 clone cells for our experiments, herein known as F1 *Mmp2* KO cells. We also used F1 Cas9^{high} no sgRNA as controls.

Cell stimulation

Im-Macs, BMDCs and BMDMs were seeded in 96 flat-bottom well plates at 200,000 cells/well for primary cells and 100,000 cells/well for Im-Macs and stimulated with 5ug/ml rhMMP2 (Enzo Life Sciences), rh-MMP9 (EMD Millipore) or vehicle control (Enzo Life Sciences); 100ng/ml of Ultrapure LPS ; Pam3CSK4, MALP2 and R848 (all from Invivogen) and 2µg/ml HMW PolyI:C (Invivogen). Cells were stimulated in Ex vivo 10 serum-free media (Lonza Biosciences, # 04-380Q) for 16-20 hours for CBA and 8 hours for RT-PCR, at 37°C.

HEK293T cell transient transfection

2.5-3x10^6 HEK293T cells (ATCC #CRL-3216, RRID:CVCL_0063) were plated in 10cm plates in DMEM with 10% FBS, and L-Glutamine and cultured overnight at 37°C before transfection with 5ug of each plasmid, using Lipofectamine 3000 (Thermo Fisher Scientific; #L3000008) at a 1:2 ratio DNA:Lipofectamine in 1ml of Opti-MEM reduced serum media (Thermo Fisher Scientific; #31985062) for 16-20 hours.

Western Blotting

HEK293T cells were collected in B150 lysis buffer (20mM Tris HCl, pH8; 150mM KCl; 10% glycerol; 5mM $MgCl_2$; 0.1% NP-40), incubated on ice for 30 minutes and centrifuged at 4°C, 14000 rpm for 16 minutes. The supernatant collected contained the protein lysates. Protein quantification was performed using a Bradford assay with a 1x dye from Biorad. Lysates were separated as described before (13). Briefly, proteins were resolved by SDS/PAGE 180-200V and transferred to PVDF membranes. Membranes were blocked using TBS/0.1% Tween-20 + 5% Milk or Bovine Soluble Albumin (BSA), probed overnight with primary antibodies and incubated with secondary antibodies for 2 hours at RT. Blots were incubated with ECL plus WB substrate (Pierce #32132), exposed to on films and developed. A complete list of primary and secondary antibodies used is in Supplemental table S1.

Co-immunoprecipitation

Lysates from cells transfected with FLAG, MYC and HA-tagged plasmids were isolated using B150 lysis buffer. 200µg of protein lysate was used for co-immunoprecipitation. Protein was incubated with anti-FLAG M2 magnetic beads (Sigma-Aldrich, #M8823), Monoclonal anti-HA agarose antibody (Sigma-Aldrich, #A2095) or Anti-c-MYC Agarose Affinity gel antibody (Sigma-Aldrich #A7470) overnight. For immunoprecipitation, samples were eluted using 3x FLAG peptide (Millipore Sigma, #4799), HA peptides (Sigma-Aldrich, #I2149) or c-MYC peptide (Sigma-Aldrich, #M2435). Samples were further analyzed using western blotting.

Bone-marrow (BM) chimeras

BM isolation: Tibia and femurs of donor mice (WT CD45.1 or *Tlr2*$^{-/-}$*Tlr4*$^{-/-}$) were collected. The tips of the bones were cut and using a 27G needle and BM flushed with RPMI, into 50ml tubes with a 70µm strainer. To make single cell suspensions, cells were passed through the strainer with the use of a syringe plunger. Cells were centrifuged and then red blood cells lysed by incubation with ACK lysis buffer (Life Technologies).

Irradiation and BM injections: CD45.1 or *Tlr2*$^{-/-}$*Tlr4*$^{-/-}$ mice were irradiated with 2 doses of 600 rads (6Gy), 4 hours apart. Mice were restrained for 1-4 minutes in a tail vein restrainer, 100ul (4×10^6 cells) of cell suspension was injected into the tail vein. 6-8 weeks post BM transfers, 1-2 droplets of blood were collected by submandibular bleeding using lancets. Blood was lysed of red blood cells and stained with an antibody cocktail mix for 30 minutes. FACS was performed to check CD45.1 versus CD45.2 engraftment and CD45.1 recipients were reconstituted with CD45.2 BM to a 83-95% purity.

Cytometry by Time of Flight (CyTOF)

3-5 x 10^6 live cells were resuspended in 1x PBS and filtered into 40µm strainer top tubes. Cells were taken for CyTOF at the Human Immune Monitoring Core facility of the Tisch Cancer Institute, Icahn School of Medicine at Mount Sinai (https://icahn.mssm.edu/research/human-immune-monitoring-center), where they were prepared for CyTOF as per the facility protocol. One lymphoid and one myeloid panel were designed, and samples were analyzed based on these. Antibodies panel and metal conjugations were generated and optimized by the Human Immune Monitoring Core facility. Supplemental table S3 contains the complete list of metal-conjugated antibodies.

RNA extraction and RT-PCR

Cells were isolated and stored in RLT Buffer with β-Mercaptoethanol or Trizol Reagent. Upon thawing, RNA was extracted with RNeasy Mini Kit (Quiagen, #74104), according to manufacturer's instructions. cDNA synthesis was performed using Sigma's ReadyScript cDNA Synthesis Mix (Sigma, #RDTR). RT-PCR was done using Qiagen's RT^2 SYBR green Master Mix with fluorescein (Qiagen, #330519).

Immunofluorescence of frozen sections

Frozen slides were washed for 10min in 1x PBS or TBS. Using a PAP pen, tissue sections were delineated. Tissues were permeabilized by incubating with 1x TBS + 0.1% Triton-X for 15min at RT, then washed with 1x TBS. Blocked the tissues with 10% BSA/TBS for 15-20min at RT in the humid chamber (or 20% serum + TBS + 0.5% BSA). Primary antibodies were prepared in the blocking buffer used and slides were incubated overnight at 4^oC. Slides were washed for 10min in TBS, RT. Secondary antibodies were prepared in 1x TBS and incubated at RT, dark for 1-2 hours. Slides were washed twice in 1x TBS and then mounted using ProLong Antifade Reagent with DAPI (Thermo Fisher Scientific, #P36931). Coverslips were added (Fisherbrand microscope cover glass, #12-545-M) and bubbles were removed by gentle pressing on the coverslips. Slides were dried in the dark and kept in the dark before imaging. Supplemental table S4 includes a list of antibodies used.

IF quantification and colocalization analysis

Quantification: Fluorescence quantification was performed using ImageJ's Fluorescence measure analysis tool (ImageJ). Several images from the tumor sections were used and 2 different tumors from each cell type were used for quantification. Briefly, from the Analyze menu select "set measurements". Select the tumor area as the region of interest (ROI) and set a fluorescence threshold to minimize background. Then select "Measure" from the analyze menu. The mean area of fluorescence will pop up on a table in a separate window.

Co-localization: Fluorescence co-localization was performed using ImageJ's colocalization plugin (Coloc2) as described in the developer's website (https://imagej.net). As described: Coloc 2 applies the pixel intensity correlation over space methods of Pearson, for scatterplots, analysis, automatic thresholding and statistical significance testing. We plotted the Pearson's coeficient values for colocalization.
